# Primate-Inspired Cooperation Emergence and Strategy Generation in Heterogeneous Robot Swarm

**DOI:** 10.34133/research.1412

**Published:** 2026-08-24

**Authors:** Bowen Wu, Renbin Xiao, Jia Zhao

**Affiliations:** ^1^ School of Artificial Intelligence and Automation, Huazhong University of Science and Technology, Wuhan, China.; ^2^ Institute of Artificial Intelligence, Huazhong University of Science and Technology, Wuhan, China.; ^3^ School of Information Engineering, Jiangxi University of Water Resources and Electric Power, Nanchang, China.

## Abstract

Biological groups exhibit an intrinsic capacity for self-organization, engendering complex and intelligent cooperative behaviors—a capability highly sought after for robotic swarms. This paper proposes an architecture for constructing swarm intelligence in cooperative tasks, termed Cooperation Emergence and Strategy Generation (CE&SG). The architecture empowers robot swarms with the ability to dynamically synthesize composite strategies through self-organized interactions, setting it apart from traditional passive or fixed approaches. CE&SG is inspired by biological observations of cooperation in primate groups, from which we extract an explanatory mechanism for self-organized strategy generation and implement it in robot swarms using a hyper-heuristic algorithm. Through this approach, we demonstrate that sophisticated swarm cooperation can emerge via continuous strategy generation. Concurrently, the framework addresses long-standing challenges in robotics, including adaptability to dynamic changes, system scalability, and strategic interpretability. In a proof-of-concept task of air defense suppression, the proposed approach outperforms several existing advanced methods. The task is executed collaboratively by robots with diverse functionalities. We tested scalability and fault tolerance mechanisms against multiple disruptive disturbances at scales of up to 250 robots and 1,500 tasks in scheduling-oriented discrete-event simulations. Physical feasibility is further validated in a high-fidelity environment with continuous kinematics and collision constraints. The evolutionary patterns of strategies are analyzed through case studies.

## Introduction

In nature, groups of insects, birds, and fish can exhibit complex and intelligent cooperation without centralized control—a phenomenon known as swarm intelligence [1]. The key to the emergence of swarm intelligence lies in the interactions among individuals, as well as their surrounding environment, leading to self-organized adaptation [2]. Inspired by this phenomenon, robot swarms have been developed and demonstrated remarkable performance in various tasks such as medicine [3], transportation [4], exploration [5], construction [6], and manufacturing [7]. Research has shown that specialized division of labor among different types of robots can enhance system efficiency, and through complementary sensing and actuation capabilities, the collective performance can exceed the sum of its individual parts [8].

However, common cooperative mechanisms employed in practical engineering scenarios may not conform to the stringent criteria of swarm intelligence. Numerous systems remain passively dependent on the centralized node to plan tasks or trajectories [9]. Although these systems may exhibit cooperative behaviors resembling swarm intelligence, their underlying mechanisms are not based on self-organization—the core characteristic of swarm intelligence. A major constraint in deploying self-organization approaches is that collective behavior emerges spontaneously following specific strategies. In practical applications, strategies in robot swarm are often predesigned before deployment. Insufficient prior knowledge of the precise conditions of the actual scenario creates a gap between the emergent behavior and the requirements. Moreover, because of the high-dimensional and complex parameter spaces involved, these predesigned strategies are difficult to adapt effectively during operation [10]. In environments characterized by numerous, multisource, and unpredictable changes, the gap between expectation and reality widens further. It poses significant challenges in safety-critical and stability-demanding scenarios, such as military, rescue, and industrial applications.

To achieve truly swarm cooperation, it may be necessary to endow robot swarms with the capability for “strategy generation”. This entails synthesizing advanced strategies by composing base strategies, enabling adaptation to diverse environments. Crucially, this process emerges through self-organization, eliminating the need for experts to possess precise a priori environmental knowledge. This capability draws inspiration from higher-order social animals. For example, many primates in nature generate strategies through social learning, such as chimpanzees (*Pan troglodytes*) and spider monkeys (*Ateles* spp.) [11]. These primate groups can flexibly fission into subgroups or fusion. Through direct and indirect interactions during this process, primates continuously gather strategies from numerous peers, then integrate them to obtain environmental feedback [12]. Through natural selection, primates in different regions have developed unique strategies [13] that precisely meet group needs, such as conflict intervention, boundary patrol, and cooperative hunting [14]. Crucially, this strategy generation is collective, self-organization, and environmentally adaptive, offering potential alignment for deployment in robot swarms as discussed herein. The biological observations above served as a design catalyst: they suggested an architecture in which strategies propagate through subgroup interactions, but the paper’s contribution rests on the engineering performance of the resulting framework. No original biological data are presented, and no contribution to primatology is claimed.

Inspired by this biological paradigm, this study proposes an architecture for constructing swarm intelligence in dynamic cooperation, termed Cooperation Emergence and Strategy Generation (CE&SG). Robots learn each other’s strategies through peer interactions. To mimic primate groups, multiple robots can autonomously establish and terminate interactions, forming subgroups that undergo fission and fusion. Through this structure, local strategies mastered by individual robots propagate throughout the entire swarm. The interaction process is continuous: once peers generate new strategies through interaction, these strategies join subsequent rounds of interaction. To implement the CE&SG architecture, we propose the primate-inspired hyper-heuristic (PIHH) algorithm, a type of automatic algorithm design technique [15]. The proposed algorithm was performance-tested on the SEAD (Suppression of Enemy Air Defenses) task for robot swarms, aiming to enable functionally diverse robots to emerge cooperative behaviors without centralized control and accomplish complex tasks.

### Explanation of the mechanisms underlying strategy generation

Based on biological observations of strategy generation in primate populations, this paper distills its mechanism into a 3-stage systemic process. This process aims to explain how strategies emerge, optimize, and ultimately form robust swarm intelligence within the CE&SG framework. It begins with a base strategy set composed of heterogeneous individuals, undergoes iterative updates through self-organization interactions and selection reinforcement, and ultimately continuously emerges environmentally adaptive strategies within a dynamic structure.

1. Initial condition

Individual heterogeneity constitutes the foundation for strategic diversification. Primate social systems exhibit pronounced heterogeneity among individuals. Based on factors such as age, gender, experience, and social rank, different individuals tend to specialize in roles they are adept at [16], leading to division of labor such as vigilance, conflict mediation, resource exploration, or infant care assistance [17]. Differences in individual performance can be understood as differences in skills, where a skill is the quality of an individual’s performance in a specific task [18] Individual heterogeneity leads to skill differentiation, providing the group with a rich, complementary initial library of strategies, which serves as the foundation for subsequent strategy combination and evolution.

Direct learning and indirect connections constitute the propagation network for strategies [19]. Primates learn new skills by observing and imitating more skilled individuals [20]. For instance, chimpanzees choose cooperation partners by observing the successful operations of others [21], and white-faced capuchin monkeys (*Cebus capucinus*) spend more time observing highly skilled individuals crack nuts. Studies on lemurs (Lemuridae) have found that knowledgeable individuals occupy more central positions in social networks. Beyond direct pathways, indirect social connections extend the reach of strategy propagation, such as through “friends of friends” [22]. These indirect links allow effective strategies to be tested and diffuse beyond the limits of direct interaction across a larger group. In PIHH, the algorithmic counterpart of these indirect connections is the fission–fusion topology’s subgroup reorganization dynamics. When strategy affinity changes trigger subgroup fusion, robots from previously separate subgroups enter the same round of Query–Key attention computation, and strategy features propagate across subgroup boundaries without explicit multihop routing. Subsequent fission events under shifted conditions further disseminate the acquired information into new subgroup configurations. For example, behavioral innovations by individual chimpanzees can spread through the group, forming shared traditions. Groups with high social tolerance are more likely to develop collective cultures, such as complex tool use [23].

2. Process mechanism

Self-organized interaction facilitates strategy exchange and diffusion. Individuals continuously exchange information during daily interactions. For instance, after discovering a new food source or water source, an individual’s movement path and foraging behavior become public information for others. Through network-level social diffusion, a strategy proven effective locally can be rapidly amplified, guiding the group toward distributed collective movement decisions [24]. Research on baboon (*Papio*) groups indicates that rather than simply following leaders, individuals pay more attention to the directions of nearby companions, achieving group movement through self-organization [25]. Similarly, members of white-faced capuchin monkey groups gradually join moving parties through imitation processes, forming a positive feedback loop where the probability of others following increases with the number of individuals already moving [26]. This process essentially entails diverse strategies receiving environmental feedback through local competition and subsequently propagating via social imitation.

Strategy selection and reinforcement are implemented based on return bias. Primate individuals continuously adjust their strategies based on behavioral outcomes. When an innovative strategy yields positive returns, the behavior is reinforced through success-biased learning [27]. Individuals show a greater tendency to imitate the behaviors of successful conspecifics, causing effective strategies to spread through the group in a snowballing effect. Research on cooperative hunting in chimpanzees confirms this: successful hunters receive more meat sharing, which in turn reinforces their cooperative alliances, allowing efficient hunting strategies to stabilize and be transmitted within the group [28]. When propagating new foraging techniques, white-faced capuchin monkeys [29] and vervet monkeys (*Chlorocebus pygerythrus*) [30] show a preference for imitating behaviors that open food boxes more quickly. In competitive task contexts, juvenile capuchin monkeys also tend to observe and learn from male individuals with higher success rates. This mechanism creates a form of selective pressure, causing strategies that yield high payoffs to be selected and reinforced more frequently within the group, while the occurrence frequency of inefficient strategies gradually decreases.

3. Evolutionary morphology

Fission–fusion-driven strategy restructuring and innovation are conducted. As resource distribution, social conflicts, or cooperative demands change, the fission–fusion nature of primate groups continuously alters the structure of interactions. For example, chimpanzee groups flexibly split into small foraging units based on the distribution of fruit trees, then reaggregate into larger groups in resource-rich areas. This structural plasticity not only reduces foraging competition pressure but, more importantly, creates diverse scenarios for self-organized interaction, facilitating differentiated strategy experimentation and cultural accumulation among individuals [31]. Analysis of the propagation network of a new foraging technique in squirrel monkey (*Saimiri sciureus*) groups found that it follows a kin-priority principle, forming a subgroup structure. Similar to individuals in fission–fusion societies, information often propagates rapidly through highly connected “hub individuals” [32]. This structural plasticity encourages different strategies to interact, be validated, and recombine within new subgroups, accelerating strategy exploration and cultural accumulation at the group level.

Continuous interaction generates strategies adapted to the environment. Under the influence of the fission–fusion architecture, base strategies are optimized through underlying interactions and then receive environmental feedback. New strategies suited to the environment are reintroduced into subsequent rounds of interaction, gradually generating more effective strategies. For example, during long-term cooperative hunting, chimpanzee groups gradually develop strategies such as driving, encircling, and capturing prey. As successful collaborations increase, individual strategies become increasingly adapted to the environment, and the degree of division of labor among individuals also rises [33]. In nut-cracking behavior among capuchin monkeys (*Sapajus libidinosus*), individuals within subgroups develop distinct methods for using hammer stones of different materials through trial and error. These localized innovations subsequently spread throughout the group via observational learning, ultimately solidifying into group-specific cultural traditions [34].

**Fig. 1.**
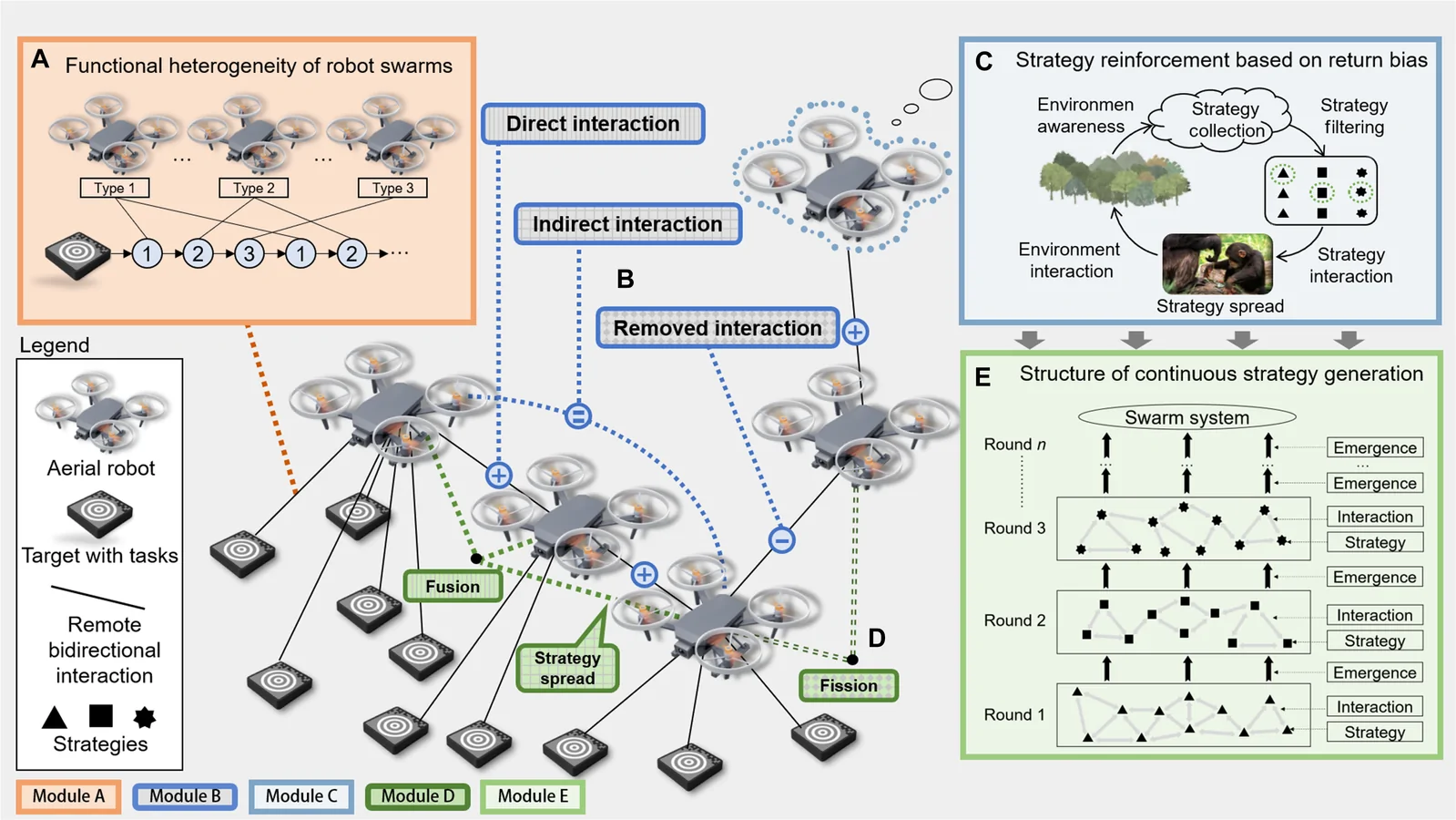
Schematic diagram of the CE&SG concept, illustrating the self-organized strategy generation through local interactions among heterogeneous robots. The framework consists of 5 functional modules: (A) heterogeneous robot swarm, (B) direct and indirect interactions, (C) selective pressure from environmental feedback, (D) fission–fusion self-organization structure, and (E) continuous strategy generation. Detailed mechanism descriptions are provided in the “Concept of CE&SG: A cooperative emergence framework for robot swarm” section. The 5 panels (A to E) describe the synthesis of decision strategies from base strategy interactions within a fission–fusion topology. SoNS [47] addresses the self-organization of communication topologies; CE&SG addresses the self-organization of decision strategies.

Figure 1E illustrates the structure of strategy generation. For clarity, assume strategy interactions persist for *n* rounds. The initial strategy set is designated as Round 1, while the final strategy set is Round *n*. Intermediate layers from Round 2 to Round *n* − 1 constitute strategies at varying levels. Horizontally, each level corresponds to a moment in time for the robot swarm, containing multiple differentiated strategies. Strategies interact with each other, triggering strategy generation. Through selection by the current environment, new strategies suited to the environment are carried forward into the next round of interaction. Vertically, the progression from lower to higher levels can be viewed as the manifestation of strategy generation and can also be regarded as the emergence of intelligence. This structure enables the swarm to adaptively generate higher-level strategies for responding to new environments through the self-organization of underlying strategies.

### Concept of CE&SG: A cooperative emergence framework for robot swarm

The core concept of CE&SG is visualized in Fig. 1. In Fig. 1A, the robot swarm in CE&SG is functionally heterogeneous, with each type of robot capable of performing a subset of tasks. Such swarms excel in complex scenarios where a single objective involves multiple task types, and system capabilities can be expanded by adding new robot types. In Fig. 1B, any robot can directly interact with others to learn their task execution strategies; it can also learn indirectly from more distant robots via intermediaries, and may autonomously terminate an interaction. In Fig. 1C, selective pressure arises during environmental interactions, filtering all strategies within the swarm. High-reward strategies are selected and reinforced more frequently, while low-reward strategies gradually diminish. In Fig. 1D, the establishment, maintenance, and termination of interactions exhibit self-organizing behavior, keeping the swarm structure dynamically evolving. This dynamic structure generates diverse local interaction scenarios, enabling different strategies to interact, validate, and reorganize within subgroups, thereby facilitating the exploration of novel strategies at the swarm level. In Fig. 1E, continuous interaction generates environment-adapted strategies. Base strategies are first refined through local interactions and then evaluated via environmental feedback. New strategies suited to the environment re-enter subsequent interactions, ultimately enabling the swarm to generate higher-level strategies best adapted to the environment through persistent interaction of base strategies.

To clarify the theoretical positioning of CE&SG, we compare it with 2 representative classes of swarm intelligence frameworks: stigmergy-based systems and multiagent reinforcement learning (MARL) for swarm cooperation. The comparison focuses on 3 core mechanisms, self-organization, strategy generation, and environmental feedback:

• Stigmergy-based systems (e.g., ant colony optimization) rely on indirect coordination via environmental modifications. While they exhibit self-organization, their strategies are typically predefined and static, lacking the capacity for online strategy generation. Environmental feedback is implicit and delayed, limiting adaptability in dynamic scenarios.
• MARL-based swarm cooperation enables agents to learn policies through trial-and-error interactions. Although MARL supports adaptive strategy acquisition, it often requires centralized training or extensive simulation, and the learned policies are usually fixed after deployment. Strategy generation is thus offline and computationally intensive, with limited interpretability and scalability in open-ended environments.
• CE&SG pursues online, self-organized strategy generation through a hyper-heuristic (HH) mechanism whose interaction structure was loosely inspired by biological self-organization. Strategies are not preprogrammed but emerge from local interactions among embodied agents, driven by real-time environmental feedback. This enables continuous adaptation, scalability, and interpretability—3 long-standing challenges in swarm robotics.

### Deployment of CE&SG: An HH algorithm based on embodied cognition

Primates can transmit and learn strategies among their peer. Regarding the origin of this capability, the traditional “Social Brain Hypothesis” emphasizes that primate brain evolution was primarily driven by social complexity [35]. However, some scholars point out that this view neglects the base role of the body and action in social cognition, subsequently proposing the “Embodied Social Brain Hypothesis”. This hypothesis posits that social cognitive abilities are built upon the interaction between the body and the environment, a process also known as embodied cognition [36]. Within the embodied cognition framework, social learning not only relies on observing and imitating others’ behaviors but also involves the integration of one’s own actions, perceptions, and environmental feedback. For instance, the discovery of the mirror neuron system indicates that primates activate their own corresponding motor representations when observing actions performed by others, facilitating imitation and the acquisition of social skills [37].

**Fig. 2.**
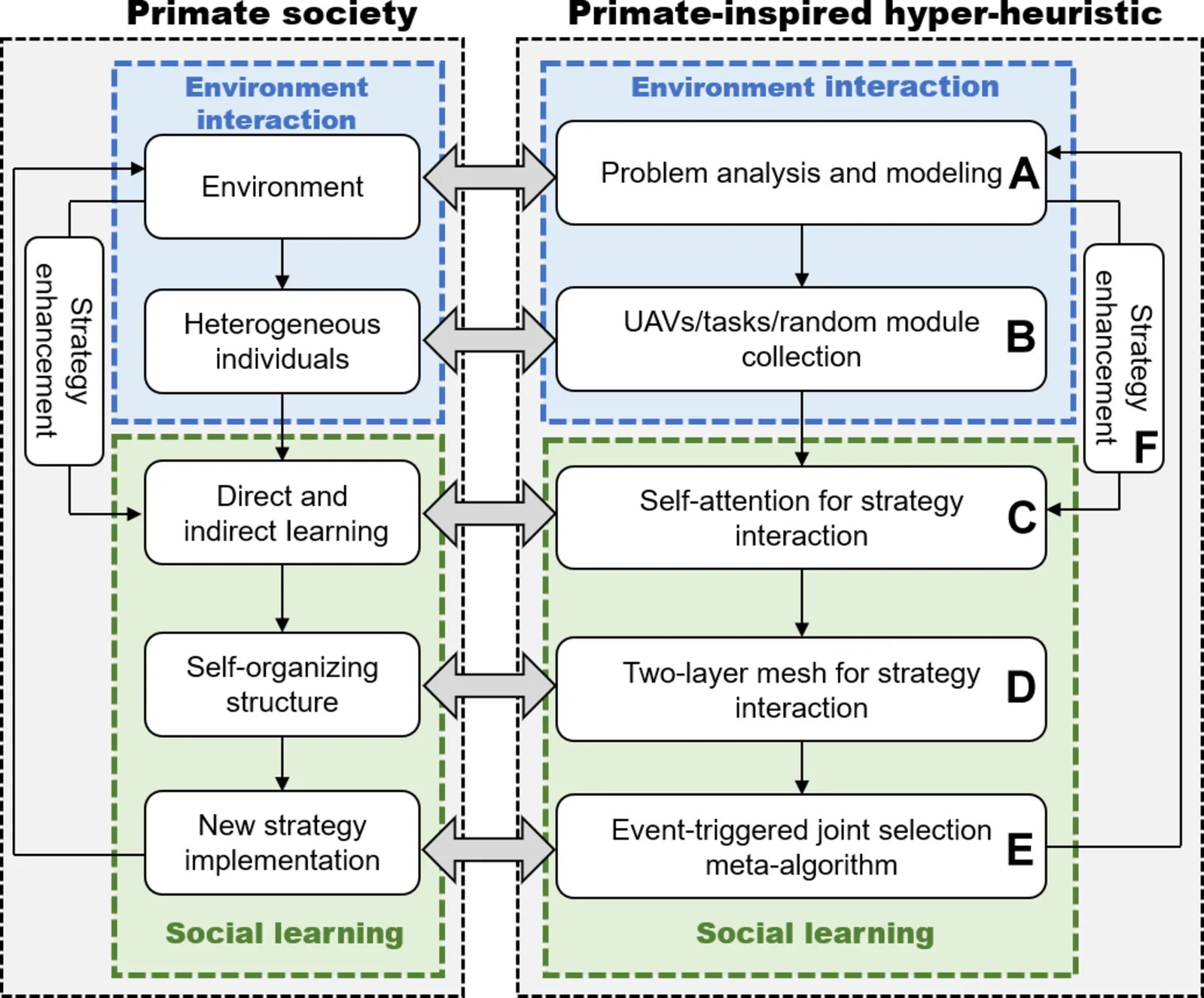
Mapping from biological inspiration to the PIHH algorithm (A to F). This figure illustrates 6 groups of mappings from primate group behaviors to the components of the PIHH algorithm: (A) environment modeling, (B) individual heterogeneity, (C) direct/indirect interactions (self-attention mechanism), (D) fission–fusion self-organization structure, (E) new strategy deployment (event-triggered joint selection), and (F) strategy reinforcement (environment-feedback-driven screening and reinforcement). Detailed descriptions of each mapping are provided in the “Deployment of CE&SG: An HH algorithm based on embodied cognition” section.

Drawing loose design inspiration from this biological process, we employ an HH algorithm. HH belongs to a class of automated algorithm design techniques driven by environment interaction. During the execution of problem instances, HH generates advanced strategies to address diverse challenges by combining base strategies. This flexible mechanism distinguishes HH markedly from traditional robot swarm planning methods, which typically employ fixed structures suited only for static problems. This paper designs an HH algorithm called PIHH that mimics primate embodied cognition processes. PIHH incorporates components such as environmental pressure, behavioral strategies, strategy objectives, and social network formation. The mapping from these biological concepts to the algorithmic components is illustrated in Fig. 2. Specifically, the problem analysis (Fig. 2A) corresponds to the primate survival environment; the feature extraction for base strategies (Fig. 2B) mirrors individual heterogeneity; the self-attention mechanism (Fig. 2C) simulates direct and indirect interactions; the 2-layer network architecture (Fig. 2D) models the fission–fusion social structure; the event-triggered selection algorithm (Fig. 2E) implements the application of new strategies; and the environmental evaluation process (Fig. 2F) reflects strategy reinforcement. The algorithm’s components and structure are detailed in Materials and Methods. This figure illustrates the conceptual mapping from biological inspiration to algorithm components; it does not imply an online/offline schedule. Training (offline, using Hebbian-like updates) and execution (online, forward-only) are separated in practice.

It is important to clarify that “strategy generation” in PIHH is not a weighted combination of fixed heuristic rules, but rather a self-organized and environment-coupled synthesis process. It differs from conventional hyper-heuristics in 3 aspects: first, strategy weights are computed in real time by a self-attention mechanism based on environmental states rather than being predefined; second, strategies form a dynamic interaction network through self-attention, where the interaction structure itself serves as the carrier of new strategies; third, through the fission–fusion structure, strategies undergo recombination and competition across subgroups, with new strategies emerging from interactions rather than preexisting. In summary, strategy generation is an environment-driven, self-organizing recombination process enabled by structured interactions.

The PIHH algorithm features the following:

• Automated strategy design. PIHH achieves the automatic generation and optimization of advanced strategies through the interaction of base strategy network architectures. This process requires no manual intervention at the strategy composition level or complex algorithm debugging; the system can screen and strengthen effective strategies based on environmental feedback, significantly reducing the engineering burden of algorithm design and adjustment.
• Dynamic environmental adaptability. PIHH simulates the iterative evolution of natural populations during training, enabling dynamic environment-driven strategy adaptation in response to factors such as sudden tasks, robot failures, and task changes. More importantly, PIHH can perceive environmental shifts, task requirements, and swarm status in real time, generating robust strategies that adapt to diverse types and intensities of disturbances.
• Scalability of problem size. Unlike common centralized preplanning methods, PIHH employs a distributed mechanism that effectively mitigates computational explosion issues arising from expanding problem scales in centralized approaches. Experiments demonstrate that PIHH maintains stable performance and low computational costs across both small-scale and large-scale tasks and swarm configurations.
• Strategy interpretability. The generated advanced strategies are dynamically composed of semantically explicit base strategies, such as distance, resource matching, and task sequencing. Engineers can understand the decision logic by analyzing strategy activation frequencies. This modular design approach enhances strategy transparency and credibility, facilitating deployment in practical systems.

To more clearly articulate the technical gaps in existing research, this paper classifies and compares current approaches to robot swarm planning. As shown in Table 1, existing methods primarily fall into 3 categories: centralized planning, distributed self-organization, and traditional hyper-heuristics. Each of these methods has limitations in terms of mechanism dependency, strategy adaptability, problem scalability, and decision interpretability. Centralized planning methods (e.g., covariance matrix adaptation evolution strategy [CMA-ES]) are heavily reliant on a central node, leading to poor robustness and limited scalability in dynamic environments. Distributed self-organization methods (e.g., democratic-centralization model [DCM]), while decentralizing control, often rely on predefined fixed rules for their cooperative behavior, making it difficult to adapt to unforeseen dynamics. Traditional HH methods (e.g., genetic programming-based hyper-heuristic [GPHH]) can combine base strategies, but the high-level strategies they generate are often fixed in structure, hard to adjust after deployment, and their internal decision logic is difficult to interpret. To address these issues, we propose the PIHH. Through a self-organized strategy generation mechanism, PIHH aims to simultaneously achieve dynamic adaptability to the environment, high scalability for large-scale problems, and strong interpretability of the decision-making process, all within a single framework for the first time.

**Table 1.** Comparison of different robot swarm planning approaches

Dimension	Centralized planning (e.g., CMA-ES)	Distributed self-organization (e.g., DCM)	Traditional hyper-heuristic (e.g., GPHH)	PIHH
Mechanism dependency	Advance allocation	Rule-based	Offline-trained, static tree structure	Online-adjustment, dynamic network structure
Strategy adaptability	None (requires replanning)	Low (rules are predefined)	Low (fixed tree structure)	High (continuous evolution)
Problem scalability	Poor (suffers from combinatorial explosion)	Moderate (performance degrades with scale)	Moderate (scales better than centralized)	High (stable performance across scales)
Decision interpretability	High (explicit optimization)	Low (implicit objectives but explicit rules)	Low (complex structures difficult to parse)	High (analyzable and traceable weight evolution)
Dynamic adaptability	None (frequent reallocation needed)	Moderate (conflicts increase with dynamics)	Moderate (only thrives in familiar environments)	High (event-triggered strategy generation)

To establish terminological precision, we provide the following operational definition of strategy generation: Strategy generation refers to the process of dynamically synthesizing composite decision strategies $S_{t}=F\left(B,\;A_{t},\;E_{t}\right)$ over a given base strategy pool $B=\left\{b_{1},\;b_{2},\;\ldots ,\;b_{18}\right\}$, through self-attention-driven structured interactions. Here, $A_{t}$ denotes the strategy interaction topology at time t (characterized by the self-attention matrix), and $E_{t}$ denotes the current environmental state. A generated strategy can be mathematically expressed as a combination of base strategies; however, the composition pattern and weights are driven by the environment in real time and emerge through iterative interstrategy interactions, rather than being predefined or learned offline. This definition clearly distinguishes PIHH from 2 classes of methods: (a) static weighting methods, where weights are fixed or learned offline and do not adapt to environmental changes; and (b) conventional hyper-heuristics, where the high-level strategy structure is fixed after deployment and cannot be adjusted. Strategy generation in PIHH is environment-coupled, online, and self-organized. A synthesized strategy is qualitatively new when it exhibits a dominant activation pattern that does not appear in the training data and cannot be obtained by interpolating between any 2 training configurations. When the environment shifts, PIHH selectively activates and suppresses different base strategies through Query–Key matching, and the fission–fusion topology allows different subgroups to converge to different dominant profiles in parallel. This is a structural reorganization of strategy participation, not a uniform rescaling of a fixed weight vector. Evidence for this claim is provided by the activation frequency divergence across environmental conditions, the topological reorganization of the strategy interaction network following unmanned aerial vehicle (UAV) failure events (see the “Case study: Patterns of strategy evolution in SEAD” section), and the emergence of leverage strategies that cannot be explained by any fixed weighting scheme.

The 6 mappings in Fig. 2 are conceptual design analogies drawn from primate behavioral observations. They are not verified mechanistic correspondences, and the paper’s empirical contribution rests on engineering performance. Table 2 documents, for each biological observation, the PIHH component it inspired, the design intuition transferred, and what is explicitly not claimed.

**Table 2.** Structured mapping from primate behavioral observations to PIHH components

Biological observation	PIHH component	Design intuition transferred	What is NOT claimed
Fission–fusion social dynamics: primate groups flexibly split and merge in response to resource distribution and social conflict [13]	Dual-layer subgroup interaction network (direct + indirect strategy interactions, the “UAV/task/random strategy set” section)	Strategy interaction should be group-local (within subgroups by affinity) rather than swarm-global; subgroup membership should be dynamic, not preassigned	That PIHH replicates primate social bonding mechanisms at a behavioral or neurological level
Social learning through observation and imitation: primates acquire skills by watching skilled conspecifics [12]	Self-attention mechanism (Query–Key–Value, the “Two-layer network interaction structure based on self-attention mechanism” section)	Peer strategy features should be weighted by their relevance to the observer’s own state; the weighting should be context-dependent, not uniform	That the self-attention mechanism models primate mirror-neuron or cognitive processes
Success-biased behavioral reinforcement: effective foraging or conflict-resolution behaviors are preferentially retained across generations [14]	Environmental feedback-driven strategy screening and reinforcement (the “Event-triggered joint selection meta-algorithm” section)	Strategy weights should be adjusted based on task completion outcomes; effective strategies persist while ineffective ones decay	That the selection pressure precisely replicates natural selection dynamics
Individual heterogeneity: primates within the same group exhibit diverse behavioral profiles and specializations [11]	Eighteen base strategies spanning 3 orthogonal dimensions (UAV × Task × Random, Table 8)	The strategy pool should cover multiple independent dimensions to ensure sufficient behavioral diversity	That the 18 strategies exhaustively enumerate all possible primate behaviors
Group-level strategy emergence: collective behaviors (e.g., cooperative hunting, boundary patrol) arise from local interactions without central coordination[13]	Strategy synthesis via attention-driven recombination within subgroups (the “Event-triggered joint selection meta-algorithm” section)	Higher-level strategies should emerge from lower-level interactions; the emergent product should not be prespecified	That PIHH captures the full complexity of primate collective intelligence
Embodied cognition: social learning depends on integration of action, perception, and environmental feedback[36]	Environment-coupled online decision cycle (event-triggered, the “Problem description and instance construction” section)	Strategy synthesis should be driven by real-time environmental state rather than offline optimization alone	That PIHH implements a computational model of embodied cognition

## Results

Simulation experiments are conducted within the context of a SEAD task. SEAD involves a chain of tasks executed by a UAV swarm to neutralize air defense systems and has demonstrated its critical role in numerous regional conflicts worldwide in recent years. Each target requires the execution of multiple, diverse task types with strict temporal constraints, such as search, location, reconnaissance, strike, and damage assessment. This necessitates precise and orderly cooperation within a heterogeneous UAV swarm to meet both functional and spatiotemporal constraints. Furthermore, in military applications, the status of the environment, targets, and the swarm itself is highly susceptible to change, rendering the problem unstructured and difficult to solve. Dynamic changes can disrupt tightly coupled cooperative plans, demanding adaptive capabilities from the swarm.

Scenario instances were generated using a previously developed discrete-event simulation system [38,39]. This system employs a parametric modeling approach, where a scenario is defined by static factors (including allocation patterns, swarm composition, sets of task attributes, and sets of UAV attributes) and dynamic factors (including type, time, location, and magnitude). Crucially, by varying just 4 key parameters—number of UAVs *N*, number of tasks *M*, dynamic intensity *P*(*D*), and the type of dynamic factors—a wide array of distinct scenario instances can be generated. This parametric modeling approach facilitates testing under highly dynamic and disruptive environmental changes, as well as ultra-large-scale swarms that are difficult to realize in practice.

The simulation is conducted in a 2-dimensional area containing multiple targets. Each target requires the sequential execution of 3 different types of tasks, performed by 3 types of functionally heterogeneous UAVs. The number of targets varied among 10, 50, 100, 200, 300, 400, and 500, with the number of UAVs set to half the number of targets. The dynamic intensity *P*(*D*) was set to values of 0.1, 0.2, 0.3, and 0.5. The types of dynamic factors included appearance of new targets, UAV failures, and task information errors, and their occurrence was configured as single-type, pairwise combinations, and random types.

To examine the stability and strategy generation capability of the proposed CE&SG framework in dynamic environments, multiple categories of experimental scenarios are designed. In the “Adaptability to environmental change” section, the type, combination, and intensity of dynamic factors are varied to evaluate system performance under disturbances. In the “Case study: Patterns of strategy evolution in SEAD” section, the evolution of the strategy interaction network is analyzed at a micro-level to observe how new strategies emerge in response to dynamic events. Together, these 2 types of experiments provide complementary evidence for the core mechanisms of the framework.

The following algorithms were selected for comparison:

• Covariance matrix adaptation evolution strategy [40]. CMA-ES is one of the most widely applied evolutionary algorithms, with use cases including CMA-ES-assisted robot optimization.
• Democratic-centralization model [41]. DCM is a distributed algorithm designed for dynamic environment and has achieved notable performance in UAV swarm planning.
• Genetic programming-based hyper-heuristic [42]. As one of the most popular HH algorithms, GPHH has been successfully applied to problems like dynamic job shop scheduling.
• Genetic programming hyper-heuristic with elitist mutation (GPHH-EM) [42]. GPHH-EM has demonstrated superior performance in integrated optimization problems involving order batching and picking path planning.
• The proposed PIHH method in this paper.

Finally, these methods were chosen because they represent 3 primary current approaches to planning robot swarms: CMA-ES as a centralized method, DCM as a self-organization algorithm, and GPHH/GPHH-EM as HH algorithms. Furthermore, these algorithms have achieved leading performance in their respective fields. Detailed problem descriptions, instance construction, and experimental settings can be found in Materials and Methods.

### Adaptability to environmental change

**Fig. 3.**
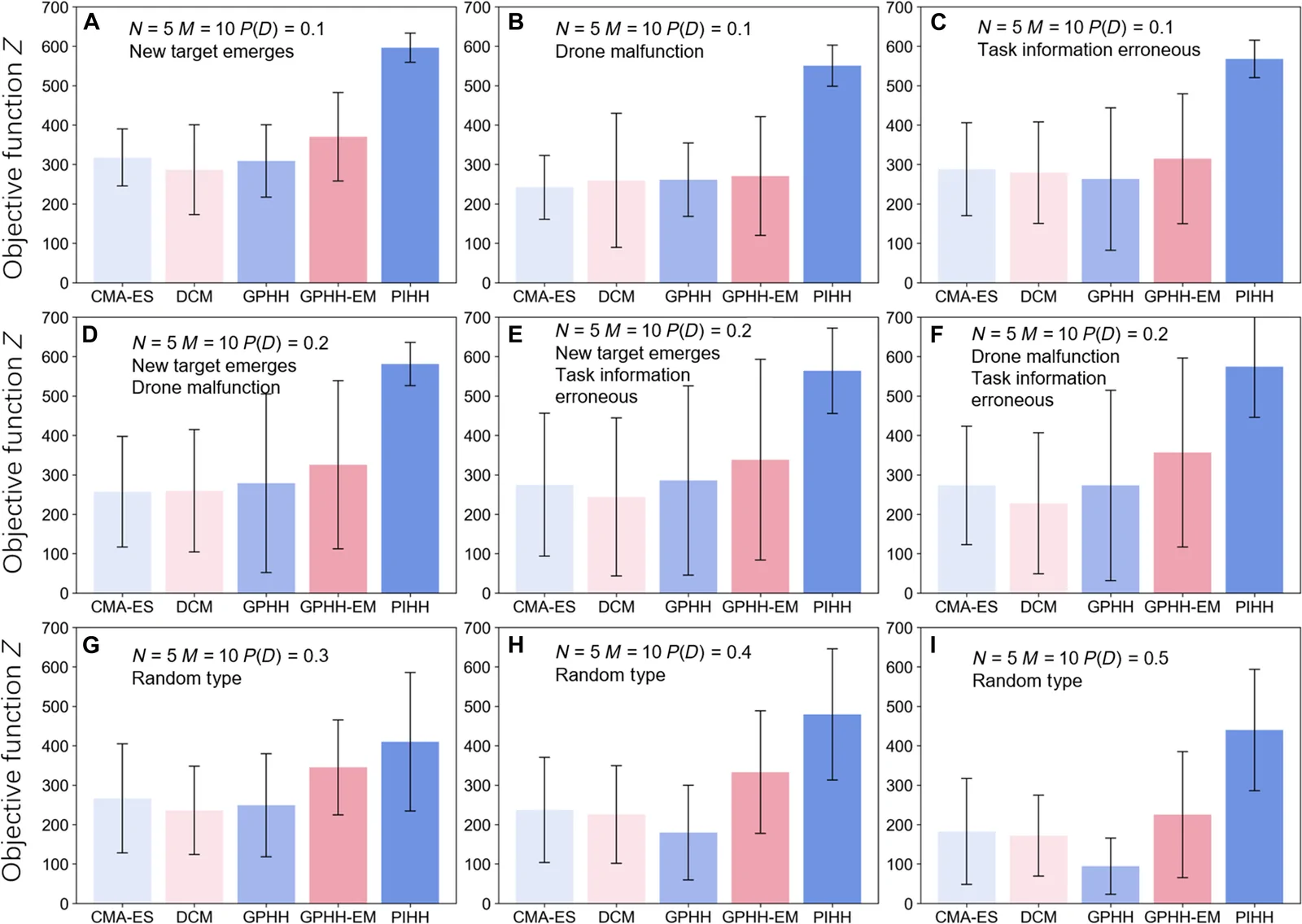
Adaptability comparison of algorithms in different dynamic environments. Dynamic factors disrupt existing cooperation schemes, and a higher degree of dynamics *P*(*D*) necessitates more frequent replanning of cooperation strategies. As task-oriented systems, UAV swarms can be evaluated using objective function values to reflect their adaptability, where a higher value indicates greater task. (A) to (C) represent simple dynamic scenarios, corresponding to 3 types of dynamic factors: the emergence of a previously unknown target, failure of a single UAV, and changes in task information of a target. In (A) to (C), the types of dynamic factors are known, but their timing and location are unknown. (D) to (F) depict more complex dynamic scenarios, combining 2 types of dynamic factors. (G) to (I) represent highly dynamic scenarios, where the number of disruptions to cooperation schemes is 3, 4, and 5 times that of (A) to (C), respectively. In these scenarios, not only the timing and location but also the types of dynamic factors are unknown. Across these 3 comparative groups, the severity of disruptions to cooperation schemes progressively increases, aiming to evaluate the robustness and recovery capabilities of the algorithms. Each scenario was independently and randomly generated 30 times to compute the mean values and standard deviations.

Figure 3 compares the adaptability of algorithms under varying dynamic conditions. The results show considerable dispersion across all algorithms, attributable to the randomized generation of most parameters of the UAVs, tasks, and dynamic scenarios. Furthermore, the varying time-window functions for calculating task reward further amplify the differences between individual algorithm runs. This diversified experimental design aims to simulate real-world uncertainties and prevent algorithms from overfitting to specific environmental conditions.

As dynamic complexity escalates across the experimental groups—Fig. 3A to C, D to F, and G to I—all benchmark algorithms exhibit performance degradation to varying degrees. The preallocated schemes generated by CMA-ES are substantially disrupted, requiring frequent task reallocations that adversely affect performance metrics. While DCM’s local interaction mechanism facilitates reallocation, extensive environmental changes also lead to frequent task conflicts. Similar environmental variations also constrain GPHH and GPHH-EM from constructing high-quality solutions. In contrast, PIHH incorporates an event-triggered joint selection meta-algorithm, enabling dynamic perception of task requirements and environmental perturbations. Its self-attention mechanism effectively captures strategy demand characteristics and matches features, generating adaptive new strategies through dynamic adjustment of attention weights, thereby achieving superior performance.

### Scalability for problem size

Scalability demonstrates an algorithm’s ability to maintain stable performance as problem size increases. Problem complexity arises from both target quantity and UAV count, with large-scale targets causing exponential growth in computational difficulty. Furthermore, task conflicts among UAVs often lead to oscillatory behavior in the swarm when UAV numbers increase. Frequent task adjustments and disorderly movements among numerous UAVs not only substantially increase overall energy consumption but also impair the swarm’s task efficiency. This study established 6 large-scale instances where the relationship between UAV count *N* and target count *M* is *N* = 0.5*M*. Research indicates that among all combinations of *M* and *N*, scenarios where *N* < *M* present the greatest complexity. The challenge lies in how to accomplish complex tasks with fewer UAVs while managing temporal relationships.

**Fig. 4.**
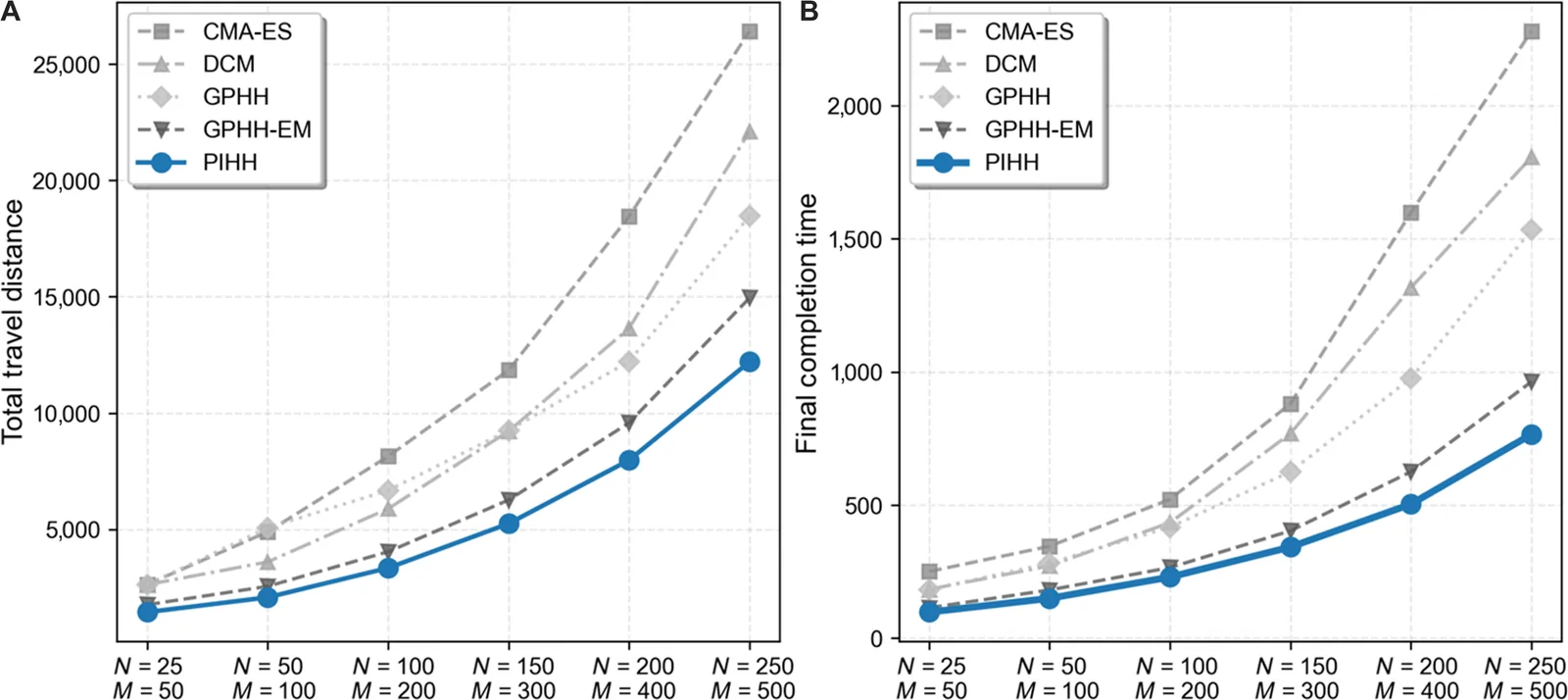
Comparison of algorithm scalability across different problem scales. Increasing problem size impacts the task efficiency and energy consumption of the swarm. (A) compares the total travel distance of all UAVs across 6 large-scale instances. Travel distance reflects the swarm’s energy consumption; a smaller distance indicates lower energy expenditure. (B) measures the time taken for all UAVs to complete the final task across 6 large-scale instances. Shorter completion times are preferable; longer times indicate suboptimal cooperative strategies. Because of temporal constraints between tasks and time window limitations, prolonged completion times suggest UAVs failed to execute tasks at appropriate times.

Figure 4 reveals that algorithm performance is comparable at smaller scales (e.g., *N* = 25, *M* = 50). As the problem scale gradually increases, CMA-ES demonstrates the greatest travel distance and completion time in most cases, such as instances (*N* = 100, *M* = 200), (*N* = 150, *M* = 300), (*N* = 200, *M* = 400), and (*N* = 250, *M* = 500). As a centralized optimizer, CMA-ES incurs substantial computational overhead in large-scale scenarios, severely compromising its feasibility for online planning and rapid response. Following CMA-ES in performance is DCM, whose travel distance increases rapidly starting from instance (*N* = 150, *M* = 300) and completion time rises sharply from (*N* = 100, *M* = 200) onwards. DCM is a distributed self-organization algorithm. Owing to the absence of global guidance, its performance deteriorates markedly when confronted with large-scale problems involving complex spatiotemporal constraints. GPHH and its variant GPHH-EM belong to HH methods, thus exhibiting overall performance superior to CMA-ES and DCM. However, the strategies generated by GPHH and GPHH-EM are static GP tree structures that become immutable once loaded to swarm. In contrast, PIHH adopts a mesh structure that enables continuous strategy optimization during runtime through weight adjustments. This allows PIHH to achieve optimal performance across all 6 large-scale instances.

### Interpretable artifacts of strategy generation

This section presents the semantically transparent decision artifacts that PIHH produces by virtue of its modular architecture—base strategies with explicit engineering semantics, inspectable attention weights, and traceable activation histories. These artifacts provide a foundation for interpretability analysis; they do not, on their own, constitute user-study-verified interpretability. The following analysis is organized around 4 concrete engineering questions: understand (what drives decisions?), predict (how does the swarm reorganize under disruptions?), diagnose (which strategies play underappreciated roles?), and verify (do the decisions respect basic engineering constraints?).

**Fig. 5.**
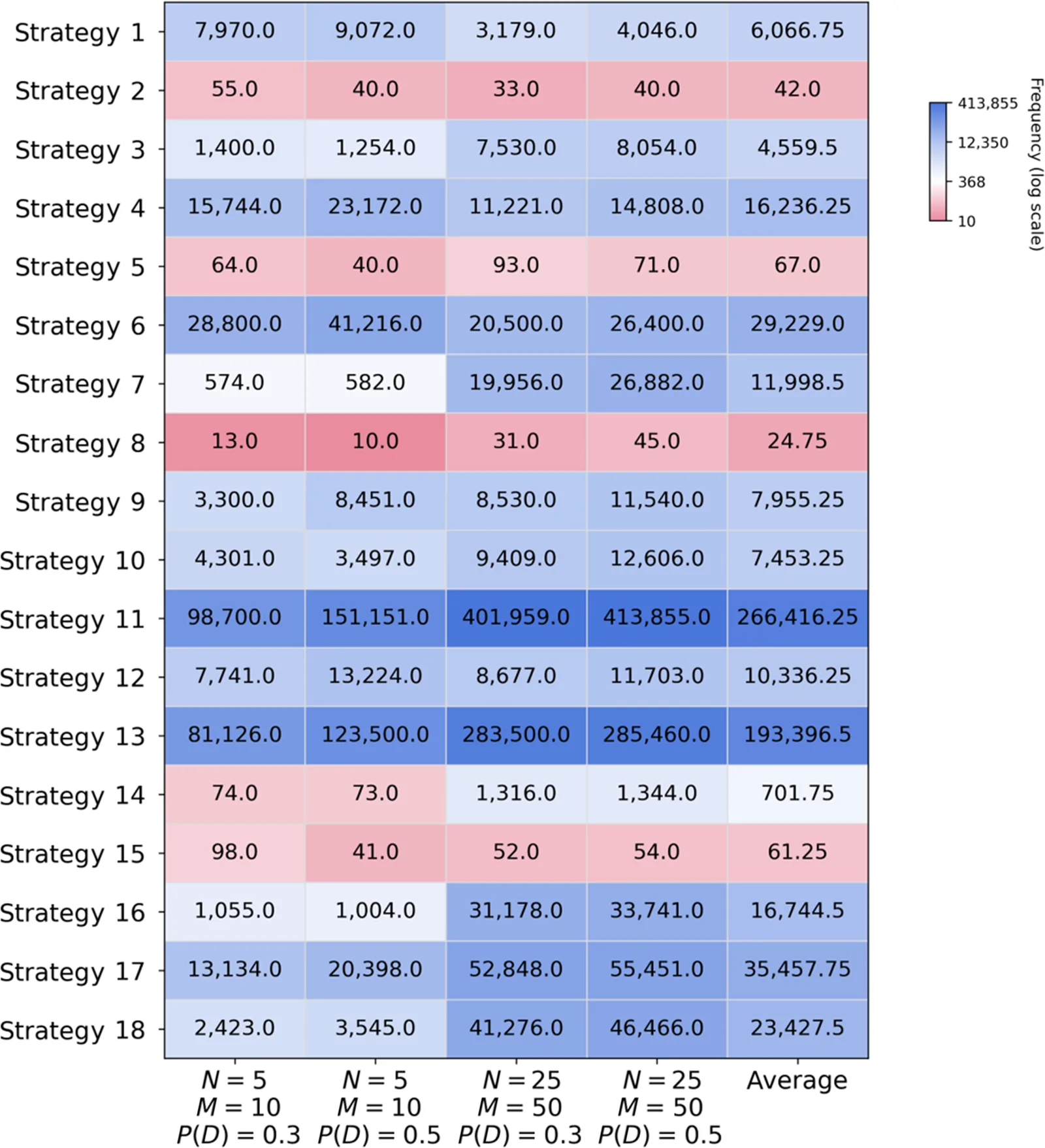
Activation frequency of strategies. During the process of designing strategies for different types of problems, PIHH activates many distinct strategies. It is assumed that the more frequently a strategy is activated in a certain class of problems, the more likely it is to play a dominant role in cooperative decision-making. Here, the vertical axis represents Strategies 1 to 18. The horizontal axis shows 4 representative scenarios and the average results, corresponding to small- and large-scale environments under strong and weak dynamic conditions, as well as the overall performance across these 4 scenarios. Colors indicate the activation frequency of each strategy: darker blue represents higher activation frequency, darker red indicates less dominance of the strategy, and white represents intermediate values.

Strategic interpretability remains a long-standing challenge in robotic swarm planning. Effective strategies are frequently highly complex, posing significant comprehension barriers for engineers. To provide a theoretically grounded interpretability analysis, we adopt a feature importance perspective widely used in explainable AI [43] and HH literatures [42,44]. Under this framework, the frequency with which a base strategy is activated serves as a proxy for its contribution to decision-making, assuming that more influential features are utilized more often (Occam’s razor principle), see Fig. 5. Therefore, by analyzing strategy activation frequencies, we reveal the internal logic and decision preferences of strategies generated by PIHH:

• From a high-frequency perspective, Strategies 11 and 13 are the most commonly used across all problems in task allocation decisions. Strategies 6, 16, 17, and 18 also show relatively high usage frequencies on average, playing important roles in decision-making. Collectively, these high-frequency strategies (Strategy 11: distance between tasks; Strategy 13: proportion of UAVs functionally suited to a task; Strategy 6: proportion of UAVs with the same function; Strategy 16: task execution sequence; Strategies 17 and 18: historical heuristic/random element) elucidate the primary decision factors underpinning PIHH’s strategy generation: intertask spatial relationships, resource-task functional matching, collaborative potential, temporal constraints, and necessary exploratory behavior. These factors align with engineering intuition, such as prioritizing spatially proximate tasks, ensuring functional compatibility, maintaining reasonable grouping, and adhering to task sequences.
• From a low-frequency perspective, the 3 least activated strategies are Strategies 5, 8, and 15, indicating their relatively lower importance in task allocation decisions. These low-frequency strategies (Strategy 5: number of UAV functions; Strategy 8: remaining resources of a UAV; Strategy 15: resources required by a task) suggest that, due to the complexity of swarm heterogeneity and task coupling, PIHH-generated strategies do not simply rely on static resource counts or function enumeration. Instead, they focus more on dynamic matching relationships and collaborative efficiency.

A deeper analysis of these observations: First, from the perspective of strategy generation, the 5 most important strategies are Strategy 6 (UAV-based), Strategies 11, 13, and 16 (task-based), and Strategies 17 and 18 (random-based). This reflects the effective utilization of the 3 designed strategy types (UAV, task, and random), with a reasonable distribution of high-frequency strategies covering major aspects of the problem. This not only supports the completeness and effectiveness of the strategy set design but also demonstrates how the effective utilization of diverse characteristics embodies swarm intelligence at the level of strategy coordination. Moreover, these strategies are self-evolved through environment-driven interactions. Second, from the perspective of strategy interpretability, each activated strategy corresponds to a clear and understandable problem feature or basic rule. Engineers can interpret the resulting complex strategy as a dynamic combination of these basic rule fragments with assigned weights. The self-organization design principle based on base strategies carries explicit engineering semantics, enhancing the transparency and explainability of the generated strategies. In addition to macro and static analysis, micro and dynamic analysis is conducted in the “Case study: Patterns of strategy evolution in SEAD” section.

### Supplementary experiment

**Fig. 6.**
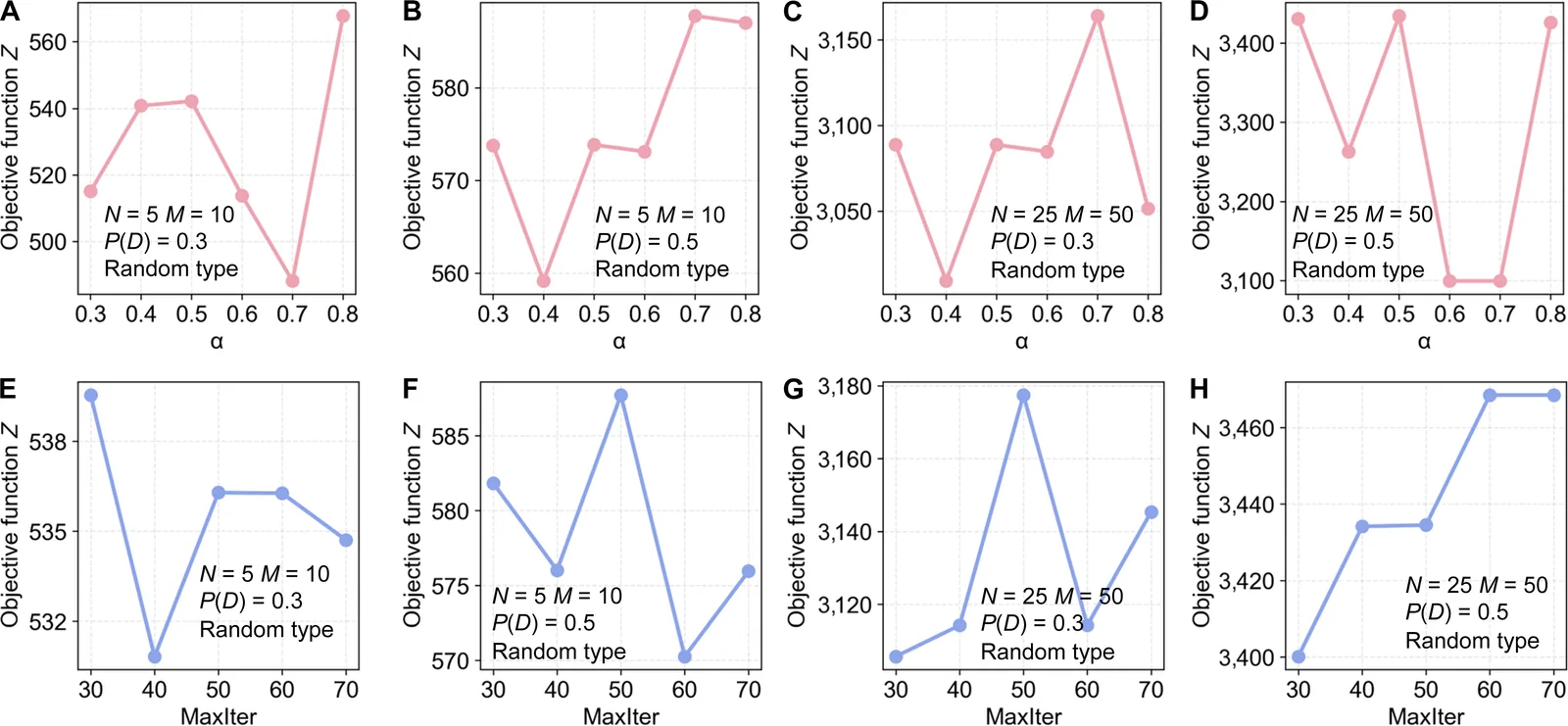
Two parameter experiments on PIHH. These experiments assess the robustness of PIHH to parameter variations. A robust algorithm reduces the engineering burden associated with parameter fine-tuning, which is particularly advantageous in dynamic problem settings. Four representative scenarios were selected, representing large- and small-scale environments under both strong and weak dynamic conditions. (A) to (D) illustrate the influence of the learning rate α on algorithm performance across the 4 scenarios, while (E) to (H) demonstrate the impact of the maximum number of interactions MaxIter, on performance under the same set of scenarios. The reason for testing these 2 parameters is that they are unique to PIHH.

The supplementary experiments focus on the unique parameters of PIHH. Specifically, we investigate the impact of the learning rate α and the maximum number of interactions MaxIter on performance. First, while the learning rate in PIHH is set to 0.5, we additionally tested other values: 0.3, 0.4, 0.6, 0.7, 0.8, and 0.9. A larger learning rate leads to greater update magnitudes for the strategy demand feature matrix *w_Q_* and the matching feature matrix *w_K_*. The experimental results of these 6 learning rate values across 4 representative environments are shown in Fig. 6A to D. It can be observed that the value of 0.5 achieves the best overall performance. Second, the maximum number of interactions in PIHH is set to 50. Experiments were also conducted with 4 other values: 30, 40, 60, and 70. As shown in Fig. 6E to H, an insufficiently small MaxIter hinders adequate interaction among strategies within the dual-layer network, leading to performance degradation in larger-scale or more dynamic scenarios. Conversely, while a larger MaxIter enhances the matching of demand features among strategies, it yields inferior results for smaller-scale problems. Overall, a MaxIter value of 50 strikes a relatively balanced performance.

It is noteworthy that even the worst performance observed in the aforementioned parameter study surpasses that of the 4 advanced algorithms compared earlier. For instance, in the instance [*N* = 5, *M* = 10, *P*(*D*) = 0.3, Random Type], the best-performing comparative algorithm (GPHH-EM) achieved a performance of 345.16, whereas the worst performance of PIHH (α=0.7) was 488.06. Similarly, in the instance [*N* = 5, *M* = 10, *P*(*D*) = 0.5, Random Type], the best comparative algorithm (GPHH-EM) achieved 224.96, while the worst performance of PIHH (α=0.4) was 558.32. This indicates that the parameter values selected in this paper yield the best overall results, and more importantly, PIHH exhibits satisfactory robustness to these 2 parameters, reducing the need for tedious fine-tuning in practical applications.

### Case study: Patterns of strategy evolution in SEAD

To conduct an in-depth analysis of the PIHH algorithm’s strategy generation mechanism in dynamic environments, this section examines a representative scenario in detail. By tracking multiple rounds of strategy interactions, it reveals the algorithm’s micro-level behavior in responding to sudden dynamics. This case study is set in a medium-scale task environment: Initially, 20 targets (*M* = 20) exist, with 10 functionally heterogeneous UAVs (*N* = 10, reconnaissance:strike:integrated=0.4/0.4/0.2) executing the task. At this scale, the interaction network exhibits moderate complexity, facilitating analysis. At simulation time *t* = 150, 2 dynamic events occur simultaneously: a new reconnaissance-type target appears in region (7, 3) (dynamic factor *d*_1_), and strike UAV U4 fails immediately due to malfunction and withdraws from the task (dynamic factor *d*_3_). This scenario tests the system’s ability to rapidly respond to unknown targets while maintaining fault tolerance and reorganization capabilities following the loss of specific functional UAVs.

**Fig. 7.**
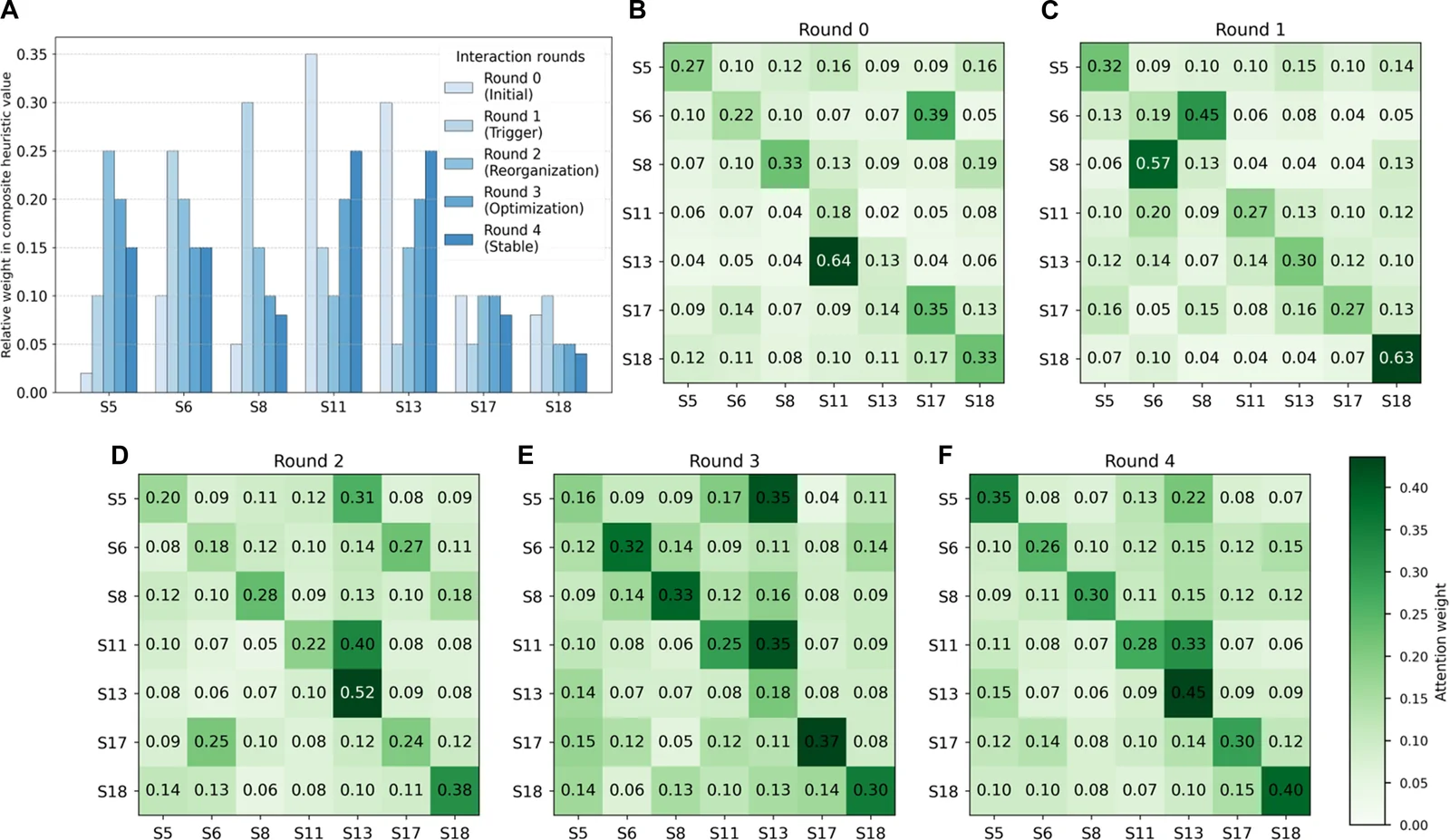
Case study of strategy evolution in a SEAD scenario: (A) Evolution of relative weights for the 7 base strategies with the most significant weight changes (>0.1) across 5 interaction rounds. The bar chart displays the relative weights of these 7 key base strategies within the heuristic values generated by the PIHH algorithm over 5 consecutive interaction rounds (Rounds 0 to 4). Weights were dynamically computed by Algorithm 1 and utilized by Algorithm 2 for joint task-UAV selection. This evolution reflects how decision logic adapts in response to dynamic events. (B to F) Heatmaps of self-attention matrices for strategies in Rounds 0 to 4, where colors represent normalized attention weights, range 0 to 1. Each heatmap represents the self-attention weight matrix for a specific interaction round. Rows correspond to the current strategy, and columns correspond to the target strategy. The color intensity in each cell indicates the normalized weight of the current strategy relative to the target strategy during heuristic value generation. These matrices visualize the dynamic interactive network between strategies, revealing how key decisions emerge, stabilize, and dissolve throughout the adaptation process.

By monitoring the activity of the strategy interaction layer in PIHH throughout this scenario, we identified 5 critical interaction rounds, spanning from the initial dynamic trigger (Round 0) to the emergence of a stabilized new strategy (Round 4). Each round of interaction was initiated by an event-triggered meta-algorithm (Algorithm 2), conducted through strategy interaction via a dual-layer self-attention network (Algorithm 1), and ultimately outputted a heuristic value for decision-making. This value essentially represents an advanced strategy dynamically weighted from multiple base strategies. Figure 7 illustrates the evolution of base strategy weights (Fig. 7A) and the dynamic reconstruction of the self-attention matrix (Fig. 7B to F) across the 5 critical rounds. The specific evolution process and decision logic decomposition are detailed as follows:

1. Initial phase (Round 0): At this stage, the swarm is in a relatively balanced state. The generated strategy is primarily dominated by high-frequency base policies, such as base strategy 11 (task-to-task distance) and base strategy 13 (proportion of UAVs matching task functionality). As shown in Fig. 7A, these policies carry the highest weight in the heuristic value. This leads to a decision logic that tends to select the spatially closest and functionally matched idle UAV for new tasks—a coordination strategy based on local optima that may lack robustness in dynamic environments. The corresponding self-attention matrix in Fig. 7B reveals a relatively concentrated strategy interaction network. Core strategy 13, as the central node, attracts the majority of attention from both core strategy 11 and itself, forming a singular decision pattern dominated by proximity.
2. Dynamic response phase (Round 1): Events *d*_1_ and *d*_3_ drastically altered the environmental landscape. The meta-algorithm detected the emergence of new target *T*_new_ and the failure of *U*_4_, triggering a new round of strategy generation. At this point, the attention weight of base strategy 8 (UAV remaining resources) surged sharply, as shown in Fig. 7A, as the system urgently needed to assess remaining strike capabilities and avoid resource conflicts. As evident in Fig. 7C, the self-attention matrix undergoes significant changes: the attention to base strategy 8 substantially increases and forms a new strong interactive connection with base strategy 6. This drives the generation of a temporary strategy prioritizing the assignment of strike tasks to UAVs with sufficient remaining resources and fewer co-functional partners. Simultaneously, owing to incomplete information, this round is accompanied by a temporary peak in the weight of base strategy 18 (random constant), prompting the system to engage in some blind exploration.
3. Rearrangement phase (Rounds 2 and 3): The system gradually converges toward the feasible solution space through multiple rounds of interaction. The role of base strategy 17 (historical heuristic value) intensifies, guiding the strategy to search near the previous round’s optimal solution. Simultaneously, base strategy 5 (number of UAV functions) is significantly activated. This is a base strategy with extremely low activation frequency in static analysis, as shown in Fig. 5. The self-attention matrices in Fig. 7D and E reveal its critical role: from Round 2 to 3, a triangular interaction pattern emerged among base strategies 5, 13, and 11, establishing a critical decision logic: when strike units are damaged, prioritize dispatching integrated UAVs to execute critical strike tasks—even if this may not represent the optimal functional match for that UAV. While this strategy appears to assign suboptimal tasks to versatile UAVs, it globally resolves strike capability bottlenecks, functioning as a leverage strategy.
4. Stable phase (Round 4): Ultimately, the system converged to a new stable strategy combination, as shown in Fig. 7F. The new strategy composition balances base efficiency (base strategies 11 and 13), functional fault tolerance (base strategies 5 and 6), and sustained exploration capability (base strategies 17 and 18). The corresponding self-attention matrix exhibits a balanced, redundant network structure: base strategy 13 and Strategy 11 remain the foundation for efficient matching, though their dominance diminishes; base strategy 5 is firmly integrated as a specialized module for handling functional deficiencies; and the interaction weights between base strategies 17 and 18 revert to baseline levels. At this stage, the swarm establishes a new cooperative paradigm: reconnaissance UAV swarms handle new target detection, while the remaining strike UAVs and integrated UAVs dynamically form teams to collaboratively execute subsequent strike and assessment tasks through functional substitution.

The structural changes observed in the strategy interaction network before and after dynamic events offer a micro-level explanation for the performance stability of PIHH under various disturbances reported in the “Adaptability to environmental change” section. From the weight evolution in Fig. 7A and the self-attention matrix changes in Fig. 7B to F, the topological structure of the strategy interaction network underwent significant reorganization in response to dynamic events. In the initial phase (Round 0), the matrix exhibited a unipolar structure centered on Strategy 13; after dynamic triggers (Round 1), new strong connections formed between Strategies 8 and 6; during the rearrangement phase (Rounds 2 and 3), a triangular interaction pattern emerged among Strategies 5, 13, and 11, establishing a new decision logic; in the stable phase (Round 4), the network evolved into a multicenter, weakly connected balanced structure. The reorganization of the network does not rely on preset rules, but is achieved through dynamic adjustments of weights and connections among strategies during runtime, illustrating how the strategy generation mechanism operates in practice.

Through a micro-level analysis of the aforementioned evolutionary process, we arrive at the following 3 findings that transcend static intuition:

• Low-frequency strategies are not ineffective. Base strategy 5 (number of UAV functions) and base strategy 8 (remaining resources) exhibit low activation frequencies under static or simple dynamic conditions (Fig. 5) and are often underestimated. However, in this dynamic scenario with missing features, the attention weights of these strategies rapidly increase after dynamic triggering, becoming pivotal hubs within the strategy interaction network (see Fig. 7C to E). They dominate critical decisions such as feature substitution and resource re-evaluation. This underscores that strategy potential must be assessed dynamically and context-dependently; static priority lists are inadequate for complex, evolving environments. This case illustrates a broader methodological point: aggregate performance metrics alone would indicate that PIHH recovered from the UAV failure, but would not reveal how the recovery was achieved. It is the per-round traceability of strategy weights (Fig. 7A) and the topological visibility of the interaction network (Fig. 7B to F) that expose the lever mechanism—Strategy 5 transitioning from a dormant state to a coordination hub. Such diagnostic insights are structurally unavailable in methods where decision logic is distributed across opaque neural network parameters (e.g., E2E-MoE, CTED-MAPPO, and DNC-A2C).
• Exploratory behavior exhibits time sensitivity. Activation of the base strategy 18 (random constant) is not uniformly distributed. It shows a distinct peak during the initial dynamic trigger phase (Round 1) in Fig. 7C, when system information is highly uncertain and requires increased exploration to escape potentially trapped invalid solution spaces. During recovery and stabilization phases, its contribution rapidly diminishes, yielding to more information-rich strategies. In this SEAD task, exploration behavior concentrates during phases of high information uncertainty. This indicates that the exploration–exploitation balance in this scenario is time-sensitive, rather than fixed at a predetermined ratio.
• Leverage strategy relies on global perception. Generated decisions underutilize one specialized UAV, yet prove efficient for the entire swarm by alleviating system bottlenecks. The emergence of such locally suboptimal yet globally efficient leverage strategies hinges on direct and indirect robotic connections in CE&SG. This mechanism enables strategies to perceive real-time global swarm states—such as the functional workload ratio reflected in base strategy 6 (proportion of UAVs with identical functions) and the spatial clustering implied by base strategy 11 (task distance), thereby facilitating collaborative decisions that transcend local fields of view.

### Comparison with recent learning based methods

The preceding experiments, conducted within a discrete-event simulation system, were primarily oriented toward validating the scheduling capabilities of the proposed algorithm under large-scale and highly dynamic problem settings. While these experiments effectively demonstrated PIHH’s advantages in adaptability, scalability, and interpretability, they abstracted away kinematic constraints and collision avoidance—factors that are critical in physically embodied robotic deployments.

**Fig. 8.**
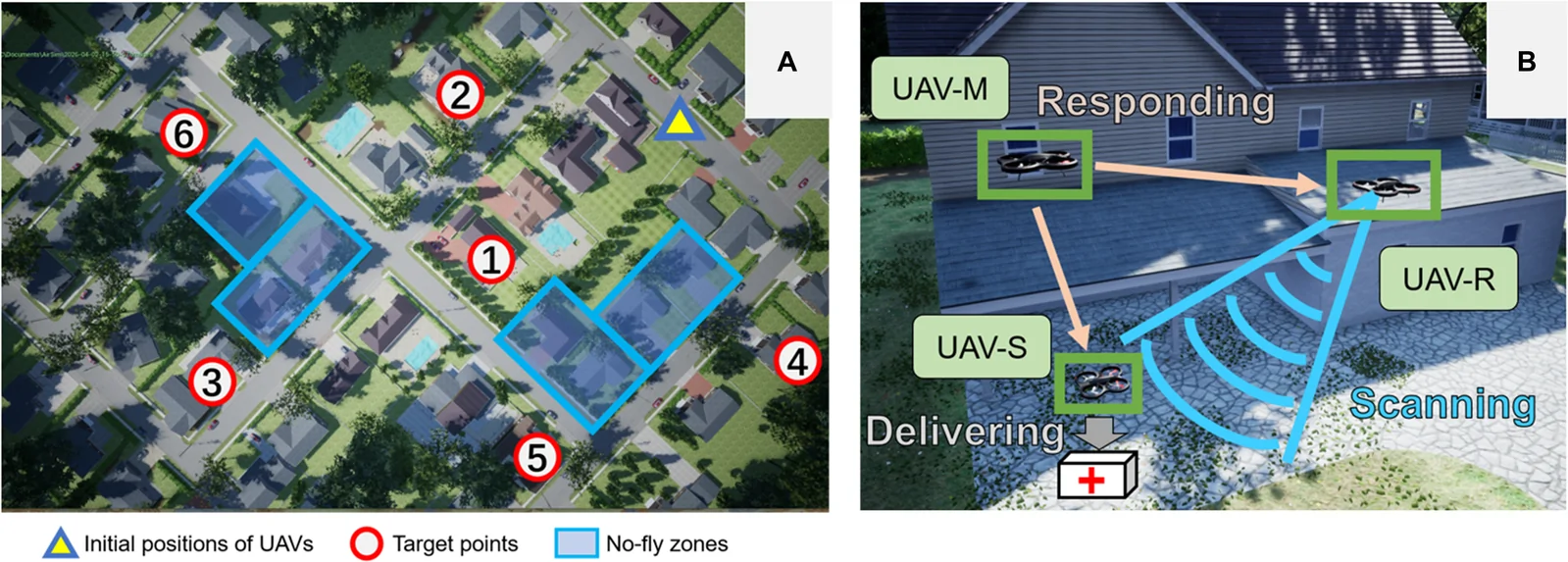
USAD high-fidelity simulation environment and mission snapshots. (A) shows a randomly generated USAD scene with *M* = 6. (B) demonstrates how 3 functionally distinct UAVs can work together.

To further substantiate the physical feasibility and cross-domain generalizability of the proposed method, we conducted supplementary experiments using AirSim, a high-fidelity physics simulator. AirSim is built upon Unreal Engine and provides 6-degree-of-freedom (DOF) rigid-body dynamics, multirotor UAV simulation, collision detection, and a suite of sensor models, thereby offering a more faithful representation of UAV behavior in complex, cluttered environments. Using the Airsim and Python API, it is straightforward to create a wide variety of simulation scenarios, as shown in Fig. 8.

The 2 experimental paradigms serve complementary validation roles in this study. The discrete-event SEAD experiments (see the “Adaptability to environmental change”, “Scalability for problem size”, “Interpretable artifacts of strategy generation”, “Supplementary experiment”, and “Case study: Patterns of strategy evolution in SEAD” sections, up to *M* = 500, *N* = 250) are designed to answer the question: Can the algorithm scale to large problem sizes under strong dynamics? The AirSim urban search and rescue (USAD) experiments (this section, *M* = 6 to 18, *N* = 0.5*M*) are designed to answer a different question: Can the algorithm operate safely in a physically realistic environment with continuous kinematics, collision constraints, and sensor feedback? The scale ceiling of the USAD experiments (*M* = 18, 9 UAVs) is determined by the computational cost of real-time 6-DOF physics simulation per UAV, not by any algorithmic limitation of PIHH. Large-scale scheduling capacity is assessed exclusively through SEAD; physical feasibility is assessed through USAD. The two are complementary, not substitutive.

The COVID-19 pandemic exposed the vulnerability of conventional urban logistics and emergency response systems under conditions of workforce shortage and strict contact restrictions. The deployment of heterogeneous UAV swarms for autonomous scanning and contactless delivery in urban search and rescue (USAD) missions has thus emerged as a compelling application domain with significant public health and societal value. For these supplementary evaluations, we selected a USAD scenario involving a heterogeneous robot swarm. Each survivor requires a sequential scan and delivery operation, necessitating coordinated deployment of scanning UAVs and transport UAVs.

To comprehensively position PIHH within the landscape of contemporary approaches, we extended our comparative analysis to include recent algorithms spanning 4 distinct methodological categories: learning-based hyper-heuristics, multiobjective hyper-heuristics, primate-inspired metaheuristics, and MARL methods. Table 3 provides a categorical overview of the comparative algorithms, highlighting their core mechanisms and primary application domains.

**Table 3.** Categorical overview of comparative algorithms

Category	Algorithm	Core mechanism	Representative application domain
Learning-based hyper-heuristic	DRL-HH [57]	Deep Q-network for online heuristic selection	Combinatorial optimization under uncertainty
Multiobjective hyper-heuristic	CBHH [58]	Compass-guided multiobjective search	Multicriteria scheduling and routing
Primate-inspired metaheuristic	RSOA [59]	Hierarchical social foraging of golden snub-nosed monkeys	Complex continuous optimization
Multiagent reinforcement learning	E2E-MoE [60]	Mixture-of-experts with end-to-end policy learning	Modular task allocation in autonomous systems
	CTED-MAPPO [61]	Centralized training with decentralized execution of PPO	Heterogeneous vehicle cooperation
	DNC-A2C [62]	Differentiable neural computer augmented actor-critic	Dynamic task offloading with partial information
	HMRTA-RL [63]	Attention-based encoder–decoder with constrained flashforward for heterogeneous coalition formation	Heterogeneous multirobot task allocation and scheduling

**Fig. 9.**
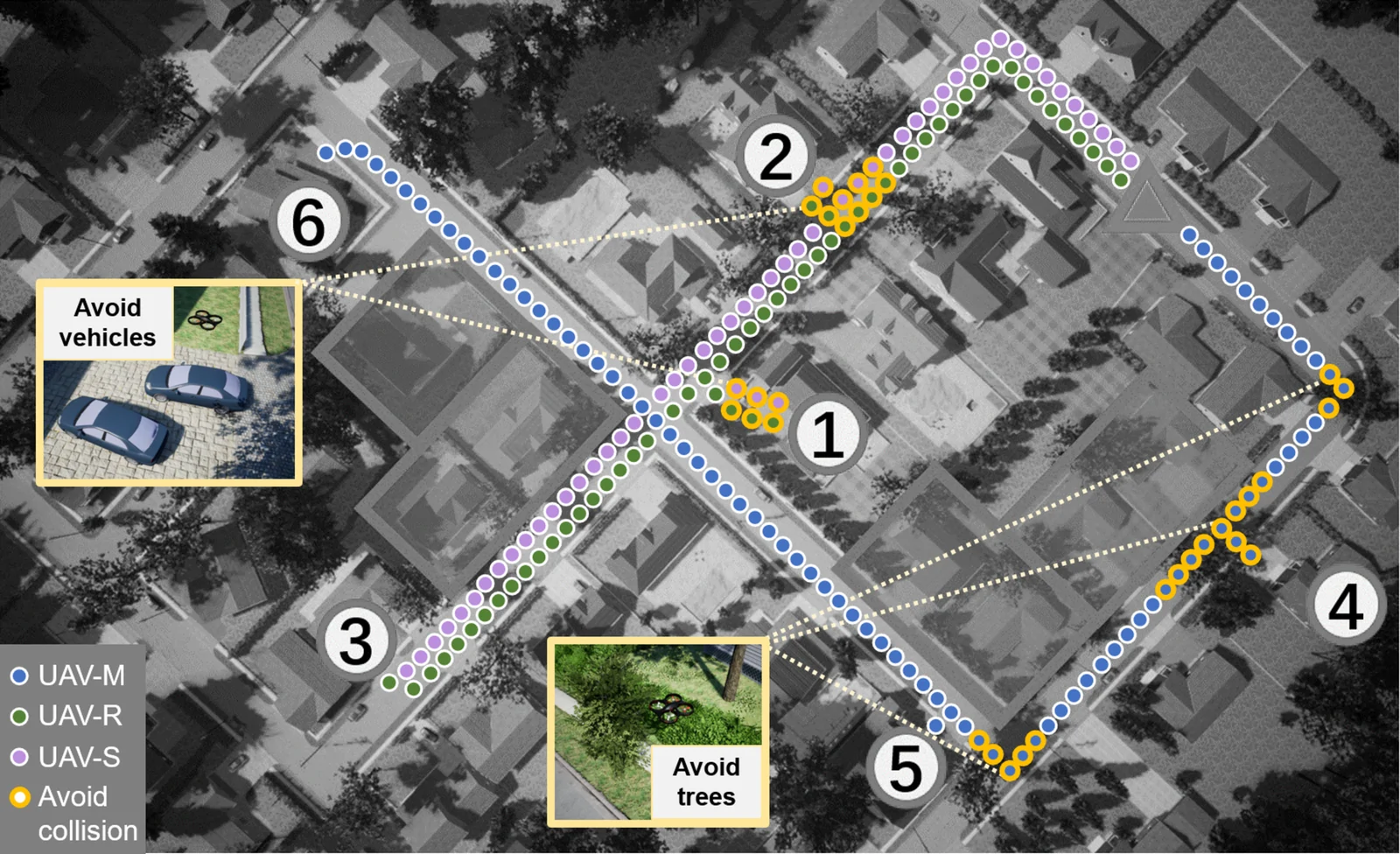
Movement paths of a PIHH-driven UAV swarm during a mission with *M* = 6.

Experimental instances were generated at 3 distinct scales, characterized by the number of survivors *M* = 6, 12, and 18, with the number of heterogeneous UAVs fixed at *N* = 0.5*M*. All algorithms were evaluated over 20 independent random runs per instance. For a clearer understanding, Fig. 9 illustrates the flight paths of a swarm of UAVs driven by the PIHH algorithm during once execution of a small-scale mission. It is clear that, due to the presence of a real-world physics engine, the algorithm must account for the effects of collisions. More broadly, Table 4 reports the average objective value and the number of collision events for each algorithm across the 3 problem scales. The objective function incorporates both task completion reward and penalties for inter-UAV collisions and near-miss incidents.

**Table 4.** Performance comparison on AirSim USAD scenarios (avg. objective value ± SD/avg. collision events)

Algorithm	*M* = 6	*M* = 12	*M* = 18
DRL-HH	687.4 ± 22.6/2.8	1,145.2 ± 34.1/6.2	1,582.6 ± 42.5/11.7
CBHH	712.8 ± 20.9/2.4	1,189.5 ± 31.7/5.5	1,634.1 ± 39.8/10.3
RSOA	468.3 ± 25.1/3.2	1,002.7 ± 37.4/7.1	1,210.4 ± 46.2/13.5
E2E-MoE	735.6 ± 23.4/3.0	1,224.9 ± 35.2/6.5	1,687.3 ± 43.7/12.1
CTED-MAPPO	758.2 ± 21.7/2.6	1,268.4 ± 33.5/5.8	1,745.8 ± 41.3/10.8
DNC-A2C	721.5 ± 24.3/2.9	1,198.7 ± 36.8/6.3	1,652.9 ± 44.5/11.9
HMRTA-RL	803.7 ± 20.1/1.9	1,339.5 ± 30.8/4.1	1,784.6 ± 38.7/6.8
PIHH(Ours)	852.6 ± 18.4/1.4	1,423.8 ± 27.6/2.9	1,865.2 ± 35.2/4.5

The results reveal several noteworthy patterns when examined through the lens of algorithmic categories:

• Learning-based hyper-heuristic (DRL-HH). DRL-HH employs a deep Q-network to dynamically select among a set of predefined low-level heuristics. In the USAD environment, this approach encounters 2 primary difficulties. First, the continuous state-action space induced by physical dynamics and collision avoidance substantially expands the Q-network’s input domain, leading to sample inefficiency and requiring extensive online fine-tuning. Second, the heuristic selection policy, once trained, remains relatively static during deployment, offering limited adaptability to unforeseen environmental perturbations. Consequently, DRL-HH exhibits the lowest objective values among all compared methods across all 3 scales, accompanied by elevated collision rates that escalate sharply as *M* increases.
• Multiobjective hyper-heuristic (CBHH). CBHH augments the HH framework with a compass-guided multiobjective selection mechanism, enabling simultaneous consideration of task efficiency and spatial distribution. While this dual-objective design partially mitigates congestion, CBHH still relies on a fixed set of base heuristics whose relative importance is determined offline. In dynamic environments where the urgency of task completion and the severity of collision risk fluctuate unpredictably, the static weighting scheme proves insufficient. CBHH achieves marginally better performance than DRL-HH but remains significantly behind PIHH, particularly in collision avoidance.
• Primate-inspired metaheuristic (RSOA). RSOA mimics the hierarchical social structure and foraging behavior of golden snub-nosed monkeys, employing a leader–follower mechanism to guide population-based search. Despite sharing a biological inspiration with PIHH, RSOA’s strategy space is confined to continuous parameter optimization rather than discrete task allocation. When adapted to the USAD problem, RSOA must discretize its continuous solutions, introducing quantization errors and losing the fine-grained coordination required for collision-free trajectory assignment. The resulting performance is the poorest among all evaluated methods, with collision rates exceeding 13 events at *M* = 18.
• Multiagent reinforcement learning (E2E-MoE, CTED-MAPPO, and DNC-A2C). These 3 MARL methods represent the current frontier in learned multiagent coordination. E2E-MoE’s mixture-of-experts architecture facilitates modular policy decomposition, CTED-MAPPO benefits from centralized training, and DNC-A2C leverages external memory to retain historical context. Despite their theoretical expressiveness, all 3 MARL approaches face a common challenge in the USAD setting: the sim-to-real gap between discrete-time training and continuous-time execution. The physical simulator introduces sensor noise, actuation delays, and unmodeled aerodynamic disturbances that are absent during policy optimization. As a result, the learned policies require substantial online adaptation, manifesting as elevated collision rates and suboptimal task completion. Notably, the performance degradation becomes more pronounced as the problem scale increases, indicating that the learned coordination strategies do not generalize robustly to larger swarm sizes.
• Heterogeneous multirobot task allocation via reinforcement learning (HMRTA-RL). HMRTA-RL employs an attention-based encoder–decoder architecture with multihead attention (8 heads, 128-dimensional embedding) and gated linear units, trained via REINFORCE with a POMO-style baseline. As the method most specifically designed for heterogeneous robot task allocation among the MARL baselines, HMRTA-RL achieves the strongest MARL performance across all USAD scales. Its attention mechanism effectively captures cross-dependencies among heterogeneous UAVs and spatially distributed tasks, and the constrained flashforward mechanism reduces deadlocks during training. However, HMRTA-RL was originally designed for 2D grid environments with constant-speed agents and does not natively account for continuous 6-DOF kinematics or collision avoidance. PIHH’s collision penalty augments [Eqs. (26) and (27)] and fission–fusion interaction structure confers additional advantages in the physically constrained USAD setting, explaining the 4.2% to 4.5% performance margin maintained by PIHH across scales.
• PIHH (ours). In contrast to the aforementioned approaches, PIHH’s CE&SG architecture offers 3 distinct advantages in physically constrained environments. First, the base strategy set is semantically grounded in real-time sensor measurements (e.g., relative distances and functional compatibility), allowing the self-attention mechanism to natively incorporate collision risk as a weighted factor without architectural modification. Second, the fission–fusion interaction structure enables local subgroups of UAVs to temporarily coordinate their trajectories, preventing the global propagation of congestion that plagues fully decentralized methods. Third, the event-triggered meta-algorithm reactively adjusts task assignments in response to near-miss events, effectively closing the loop between perception and replanning. As evidenced in Table 4, PIHH achieves the highest objective values across all scales while maintaining the lowest collision counts. The performance gap widens with increasing *M*, underscoring the scalability of the self-organizing strategy generation mechanism.

In summary, the comparative analysis across multiple algorithmic categories confirms that PIHH’s biologically inspired, self-organizing strategy generation framework confers tangible benefits in realistic, physics-constrained swarm missions. Its ability to integrate perceptual feedback, adapt to dynamic disturbances, and scale to larger swarm sizes distinguishes it from both traditional hyper-heuristics and contemporary MARL methods.

### Ablation study: Necessity of fission–fusion topology

To rigorously determine whether the biologically inspired dual-layer fission–fusion interaction structure constitutes a necessary mathematical component or merely a narrative device, we conducted an extensive ablation study in SEAD. This study compares the original PIHH algorithm against 2 variant architectures stripped of this specific topology. The first variant, termed PIHH-NoFission, removes the hierarchical dual-layer structure entirely, placing all 18 base strategies within a single flat layer that interacts via a fully connected self-attention mechanism. The second variant, termed PIHH-Transformer, replaces the entire fission–fusion network with a standard Transformer encoder block, incorporating multihead self-attention and a position-wise feed-forward network. This allows us to assess whether a generic, state-of-the-art attention architecture can replicate the benefits of the specialized biological structure.

The evaluation was conducted across 6 distinct environmental configurations to ensure the generalizability of the findings. These configurations varied the problem scale, represented by the number of survivors *M* (with the number of UAVs *N* fixed at 0.5*M* to maintain consistency with our main scalability tests), and the dynamic intensity *P*(*D*), which governs the frequency of unpredictable events such as UAV failures or new task appearances. The specific settings spanned small (*M* = 20), medium (*M* = 50), and large (*M* = 100) scales under both low dynamic intensity [*P*(*D*) = 0.2] and high dynamic intensity [*P*(*D*) = 0.4]. Each configuration was executed for 30 independent random runs, and the average objective value along with the standard deviation was recorded.

The comparative results, detailed in Table 5, unequivocally demonstrate the necessity of the fission–fusion topology for achieving robust and scalable performance. Across all 6 scenarios, the original PIHH consistently outperforms both ablation variants, with the performance gap exhibiting a clear and interpretable pattern. The most striking observation is the behavior of the PIHH-NoFission variant under high dynamic stress. In the large-scale, high-dynamic scenario [*M* = 100, *P*(*D*) = 0.4], its performance collapses to an objective value of 1,329.5, representing a 23.7% deficit compared to the 1,742.5 achieved by PIHH. This deficit is substantially more severe than the 14.2% gap observed in the corresponding low-dynamic scenario [*M* = 100, *P*(*D*) = 0.2]. This divergence indicates that without the structured, temporary subgrouping enabled by the dual-layer fission–fusion process, the interaction network is highly susceptible to a “winner-take-all” dynamic, where a small number of high-frequency strategies rapidly dominate the attention matrix and suppress the timely activation of critical but low-frequency strategies needed for fault recovery and adaptation.

**Table 5.** Ablation study of fission–fusion topology (avg. objective value ± SD)

Scenario	PIHH (fission–fusion)	PIHH-NoFission (single-layer)	PIHH-Transformer (standard arch)
*M* = 20, *P*(*D*) = 0.2	653.4 ± 18.2	571.7 ± 19.7 (−12.5%)	606.4 ± 18.4 (−7.2%)
*M* = 20, *P*(*D*) = 0.4	538.7 ± 24.1	438.5 ± 23.1 (−18.6%)	477.3 ± 21.4 (−11.4%)
*M* = 50, *P*(*D*) = 0.2	1,102.6 ± 22.8	944.9 ± 24.9 (−14.3%)	1,007.8 ± 20.8 (−8.6%)
*M* = 50, *P*(*D*) = 0.4	948.9 ± 31.5	748.7 ± 28.2 (−21.1%)	817.0 ± 27.1 (−13.9%)
*M* = 100, *P*(*D*) = 0.2	1,987.3 ± 35.2	1,705.1 ± 36.1 (−14.2%)	1,808.4 ± 32.0 (−9.0%)
*M* = 100, *P*(*D*) = 0.4	1,742.5 ± 40.8	1,329.5 ± 39.0 (−23.7%)	1,479.4 ± 34.6 (−15.1%)

In contrast, the PIHH-Transformer variant exhibits a degree of robustness against this collapse, yet its performance still lags behind the original PIHH by a significant margin ranging from 7.2% to 15.1% across the tested scenarios. While the generic Transformer architecture provides a more complex and expressive mechanism than a single fully connected layer, its reliance on a static global attention pattern proves less effective than the dynamically reconfigurable subgroups inherent to the fission–fusion model. The biological mechanism of groups splitting and merging based on local context appears to furnish a superior inductive bias for this class of distributed task allocation problems, enabling a more nuanced balance between local exploration of task assignments and global optimization of swarm efficiency.

The experimental results show consistent patterns across independent tests. Performance recovery after dynamic disturbances (Fig. 3), stable scalability across 6 problem scales (Fig. 4), and convergence within a few interaction rounds (see the “Case study: Patterns of strategy evolution in SEAD” section) indicate that strategy configurations reliably restabilize after perturbations. The activation frequency divergence across conditions (Fig. 5), the topological reorganization of the strategy interaction network after a UAV failure (see the “Case study: Patterns of strategy evolution in SEAD” section), and the leverage strategy phenomenon (Discussion) demonstrate that the system produces different dominant strategy profiles under different environmental conditions. The “Theoretical property discussion” section provides theoretical characterizations complementary to these empirical observations.

## Discussion

This paper has sought to equip robot swarms with a capacity—self-organized strategy generation—that natural collectives exhibit but engineered systems have long lacked. Beyond the specific performance gains reported above, our results suggest that self-organized strategy synthesis through local interactions constitutes an alternative design direction for building robot swarms, distinct from both centralized preplanning and offline-trained policies. The consistent advantages observed across dynamic adaptability, scalability, interpretability, and generalization—4 long-standing challenges in swarm robotics—represent consistent performance gains across multiple dimensions. Collectively, they indicate that strategy generation through embodied, self-organized interaction is a promising design framework for robot swarms. We do not claim to establish a new theory of biologically inspired swarm intelligence. Rather, this paper demonstrates that incorporating bio-inspired self-organizing interaction structures into HH algorithm design can yield substantial and consistent performance advantages across dynamic, scalable, and heterogeneous task environments.

### Physical deployment transition

The AirSim USAD experiments (see the “Comparison with recent learning based methods” section) validated PIHH under continuous kinematics and collision avoidance. These are motion-planning-level constraints; the following discussion addresses 3 decision-level constraints not covered by AirSim: communication delays, onboard computational limitations, and residual sensor noise.

• Communication delays cause the self-attention mechanism to compute weights from outdated peer strategy vectors. This misaligns Query–Key matching: UAV *i* evaluates its strategy affinity against UAV *j*’s prior state, potentially inducing oscillatory weight reassignments if *j* has since changed its strategy profile. In the fission–fusion topology, staleness can induce erroneous subgroup joining or delayed fission, and convergence time increases with delay because subgroup consensus requires communication round-trips. Above a critical threshold, the swarm may remain in a perpetual transient state. As a concrete mitigation, we propose a decentralized bounded-staleness synchronization policy: each UAV attaches a local timestamp to broadcast vectors and, upon reception, computes an age-dependent confidence weight that exponentially decays stale peer contributions before Softmax normalization. Features exceeding a maximum staleness threshold can be supplemented with linear extrapolation from recent history. This scheme is fully decentralized and introduces negligible computation overhead.
• On resource-constrained platforms, the per-event pipeline may exceed the interevent interval under high event rates, creating a backlog of decisions that operate on stale state. Stale decisions reduce swarm efficiency, triggering more events and further backlog. To mitigate this, the pipeline can be partitioned into 2 tiers. The critical tier (per-event) handles candidate strategy feature evaluation, *K* × *K* attention, heuristic scoring, and task selection. The background tier (asynchronous) handles global strategy weight recalibration, subgroup membership evaluation, and exploration budgeting. Newly triggered events interrupt background computation, bounding critical-tier latency. An adaptive mode can temporarily promote strategy recalibration to the critical tier during rapid environmental transients.
• Residual sensor noise propagates to distance-based features (S1 to S3, S11, and S12) in proportion to position estimation error. Uncorrelated random noise across UAVs is partially averaged out by Softmax relative normalization over multiple decision cycles. Correlated noise, such as Global Positioning System (GPS) multipath, is more consequential: a coherent position bias across the swarm can systematically distort attention weight distributions. An extended Kalman filter fusing inertial measurement unit (IMU), visual odometry, and GPS can mitigate this as a preprocessing module without modifying PIHH. A robust attention variant with sublinear scaling of outlier features is a direction for future algorithmic work.

Each mitigation strategy is compatible with PIHH’s distributed, event-driven architecture. These constraints can be simulated within the existing discrete-event framework by injecting artificial delay, bounding computation time, and adding controlled noise, to characterize PIHH’s sensitivity to each constraint before physical deployment.

The deployment feasibility of the complete decision pipeline—strategy feature extraction, self-attention computation, heuristic scoring, and task selection—on resource-constrained embedded platforms and the quantitative performance relationship between embedded and workstation hardware environments await further characterization through future hardware-in-the-loop validation experiments.

### Automation scope and strategy discovery

PIHH automates strategy synthesis at the composition layer but not at the vocabulary layer. The 18 base strategies were manually designed by enumerating decision heuristics along 3 dimensions—agent status, task characteristics, and exploratory behavior (see the “UAV/task/random strategy set” section). Once defined, all subsequent operations proceed without manual intervention. The following discusses the scope of this constraint, cross-domain transferability, and pathways toward greater autonomy in strategy discovery.

• Scope of the constraint. Manual base strategy design is a one-time per-problem-class investment following a principled decomposition template, distinct from per-instance parameter tuning. The SEAD-to-USAD portability (see the “PIHH deployment to the USAD mission” section) shows that the vocabulary can be reused when domains share task model and spatial semantics. When domain semantics diverge substantially, the vocabulary requires reidentification.
• Cross-domain transferability: limitations and compensatory directions. The current base strategy set was designed through “3-dimensional decomposition, per-dimension enumeration”. The decomposition template—agent status, task characteristics, and exploratory behavior—is domain-agnostic, but it specifies only the dimensional framework, not what to place within each dimension. When the target domain’s task semantics diverge substantially from SEAD—e.g., area coverage lacks a discrete “task completion” concept, whereas warehouse logistics closely resembles SEAD—decision heuristics in that dimension must be reidentified. The SEAD-to-USAD migration succeeded with only semantic reinterpretation precisely because the 2 domains happen to be semantically isomorphic on this dimension—a favorable special case, not a general capability. Two directions may reduce the manual design burden. First, per-dimension strategy enumeration can be formalized as a set of guiding questions, turning creative design into guided completion. Second, a large language model (LLM) prompted with the domain description and guiding questions can propose candidate heuristics; the proposed heuristics are screened through lightweight simulation, and the human role shifts from proposer to reviewer. Neither direction eliminates manual involvement; both reduce the per-dimension design cost.
• LLM can assist in base strategy design. Given a target domain description and the decomposition template, an LLM can propose candidate heuristics drawing on patterns learned during pretraining. A retrieval-augmented generation component can further ground these proposals against validated robotic planning literature, reducing the risk of generating strategies untethered from practical constraints. After candidate strategies are screened through lightweight discrete-event simulation—eliminating variants that violate battery constraints, kinematic limits, or causal logic—surviving strategies can be fed back to the LLM to refine subsequent generation, forming an iterative improvement loop. This pipeline faces 2 practical difficulties requiring sustained effort: some LLM-generated strategies may be superficially plausible but physically infeasible and must be rigorously filtered in simulation; and the structured corpus of validated heuristics that retrieval-augmented generation depends on is currently at an early stage of development. On a longer time scale, base strategies could be modeled as learnable parametric functions and optimized concurrently with attention weights, forming a meta-learning loop over the strategy space. We note that the following kinds of tacit knowledge in base strategy design resist automation in the foreseeable future: kinematic intuitions about specific hardware platforms, experiential judgment about nonlinear interactions among multiple constraints, and recognition of boundary conditions not explicitly modeled in the task semantics. The realistic near-term goal, therefore, is not to eliminate human involvement but to shift the human role from direct strategy designer to reviewer and validator guided by the decomposition template.

The adaptive stage-switching principle in tri-population evolutionary optimization [45]—dynamically reallocating computational budgets across subpopulations based on convergence and diversity metrics—can inform PIHH’s multiscenario training. Instead of uniform resource allocation across E1 to E3, the training budget can be directed toward the environment type where the current population performs worst, reducing total training cost without degrading generalization.

### Strategy collapse prevention

Global Softmax can drive attention distributions toward single-strategy dominance: a high-scoring strategy is exponentially amplified, compressing the weight space of others. The fission–fusion topology counteracts this through subgroup-level Softmax partitioning. The following presents causal (ablation), process (weight trajectories), and quantitative (entropy) evidence.

Subgroup formation via cosine similarity clustering partitions the global Softmax into independent local operations. Each subgroup can converge to a different dominant strategy profile, and these intergroup differences are preserved rather than averaged. When the fission–fusion structure is removed (PIHH-NoFission), performance degrades by 23.7% under high-dynamic, large-scale conditions. PIHH-Transformer, with multihead attention but without subgroup partitioning, degrades by 7.2%–15.1%.

The weight trajectories documented in the “Case study: Patterns of strategy evolution in SEAD” section provide direct process evidence. S5 (UAV functional count) is nearly dormant in static analysis (Fig. 5), with a pre-event weight of 0.02; following the UAV failure event, its weight rises to a peak of approximately 0.25 in Round 2 and stabilizes at roughly 0.15 at convergence. This transformation—from a marginal strategy to a sustained portfolio member—would be structurally precluded under global Softmax, where the dominance of initially high-weight strategies prevents such a re-ranking. S8 (residual resource assessment) surges to 0.30 during the Round 1 emergency window and then declines to 0.08, matching its functional role as an emergency evaluator rather than a long-term heuristic. S18 (random exploration) undergoes brief Round 1 mobilization and then decays below baseline, consistent with transient exploration triggered by information insufficiency. The 3 trajectories differ in direction, magnitude, and duration. Under global Softmax collapse, one would expect all strategies to converge toward the dominant one; the differentiated trajectories contradict this prediction.

**Fig. 10.**
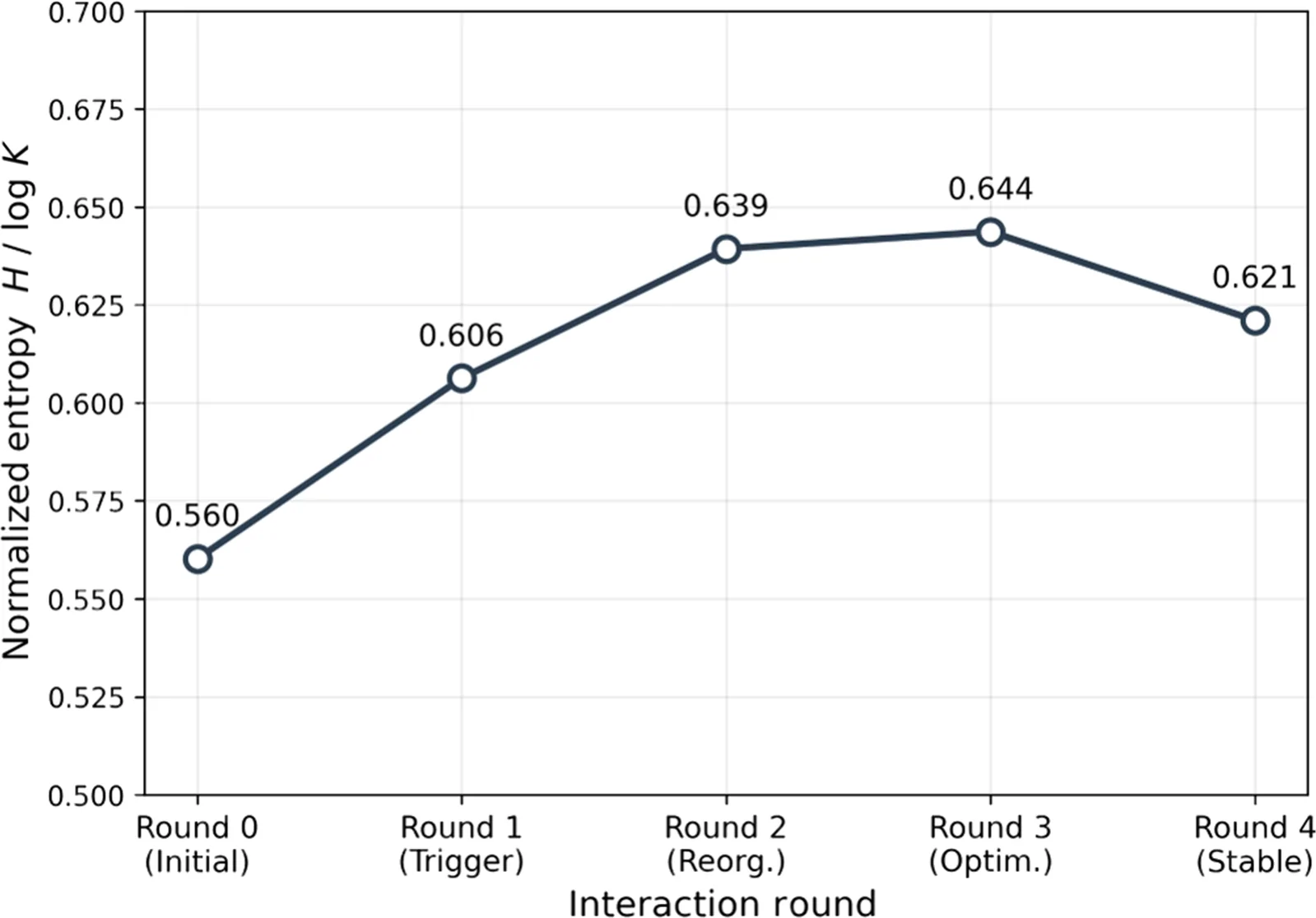
Normalized strategy weight entropy change trajectory.

Normalized entropy (*H*/log*K*) rises from a baseline of 0.560 to a peak of 0.644 (+15.0%) and settles at 0.621 (+10.9%), see Fig. 10. The trajectory—rise, peak, and stabilization above baseline—contradicts the monotonic decline predicted by collapse. The entropy increase coincides with S18 activation (Rounds 1 and 2), indicating the system broadens strategy participation under uncertainty and contracts to a more diverse configuration than the pre-event baseline. Within the tested conditions, the fission–fusion topology prevents strategy weight collapse. Strategy components reorganize differentially rather than converging uniformly.

The coupled relationship between strategy weight distribution and subgroup topology parallels the mutual reinforcement principle in feature selection [46], where soft pairwise weights and hard cluster assignments are jointly optimized. In PIHH, attention-based strategy synthesis and fission–fusion subgroup partitioning engage in a similar co-evolution dynamic.

### Limitations of bio-inspired design methodology

The biological mapping in Table 2 is based on interpreted behavioral patterns rather than quantitative field data. Two directions could strengthen the biological grounding, though both require interdisciplinary collaboration beyond the current scope: parameterizing fission–fusion dynamics against empirical primate field data, and systematically comparing PIHH’s performance with architectures inspired by alternative biological models (e.g., insect eusociality and cetacean pod dynamics).

### Comparison with self-organizing nervous systems

CE&SG and Self-organizing Nervous Systems (SoNS) [47] both employ self-organized hierarchical structures, but at different layers: SoNS addresses communication topology, and CE&SG addresses decision strategy synthesis.

SoNS constructs communication topologies through quality-based recruitment competition. Robots exchange recruitment messages; the higher-quality robot becomes parent, forming a directed tree rooted at a brain. The brain sends actuation commands downstream. SoNS demonstrates that robots can autonomously establish and reconfigure multilevel communication architectures.

CE&SG/PIHH synthesizes task allocation strategies through self-attention. Each robot uses its own strategy feature vector as Query and peer vectors within its subgroup as Keys and Values. Subgroup membership is determined by cosine similarity clustering of strategy features. The CE&SG hierarchy describes the progressive abstraction of strategic knowledge: base strategies interact, recombine, and produce synthesized strategies in response to environmental feedback. CE&SG demonstrates that decision strategies can be adaptively synthesized from a predefined vocabulary through a self-attention mechanism.

Table 6 provides a systematic comparison of the 2 frameworks across 13 dimensions, including core problem, layer of operation, core mechanism, group formation method, nature of the leadership entity, source of biological inspiration, nature of hierarchy, form of system output, post-deployment adaptability, offline training requirement, scalability, fault tolerance, and real-robot validation.

**Table 6.** Systematic comparison between CE&SG/PIHH and SoNS

Comparison dimension	SoNS [47]	CE&SG/PIHH (this work)
Core problem	Self-organization of communication topology and system architecture	Adaptive synthesis of task allocation strategies
Layer of operation	Communication/control architecture layer	Decision/strategy layer
Core mechanism	Recruitment competition → tree topology	Self-attention → strategy weights
Group formation	Explicit bidirectional negotiation (quality bidding + accept/reject)	Implicit strategy affinity clustering (cosine similarity)
Leadership entity	Brain robot (explicit supervisory authority, sends control commands)	High-attention strategy (no supervisory authority, influences weight distribution)
Biological inspiration	Mergeable nervous systems (MNS)	Primate fission–fusion societies
Nature of hierarchy	Parent–child connectivity in the communication topology	Progressive cognitive hierarchy from base strategies to synthesis
System output	Connection topology + control commands	Strategy weight distribution + task selection
Post-deployment adaptation	Topology reconfigurable (split/merge/redistribute)	Strategy weights adaptively recalibrated (attention redistribution)
Offline training required	No (rule-driven)	Yes (multiscale evolutionary training of attention weights)
Scalability	250 robots, establishment, and convergence time	250 robots, task completion rate, and computation time
Fault tolerance validation	≈0.6 permanent failure + 30 s global communication/vision outage	UAV failure + dynamic task reallocation
Real-robot/physical validation	8 heterogeneous aerial-ground robots (4 missions)	5 UAVs in AirSim physics simulation (continuous kinematics + collision avoidance)

The 2 frameworks are complementary. SoNS provides communication infrastructure; PIHH provides decision strategies that operate within that infrastructure. A complete system could adopt SoNS for communication topology and PIHH for task allocation decisions within each SoNS substructure.

Direct empirical comparison on identical scenarios is methodologically constrained because the 2 frameworks parameterize their evaluations differently. SoNS evaluates communication topology robustness under varying robot density, communication range, and failure rate. PIHH evaluates decision strategy adaptability under varying task count, UAV composition, temporal coupling, and dynamic event types. A scenario that is meaningful for one would strip core variables from the other. The 13-dimension comparison in Table 6 provides a conceptual alternative to head-to-head benchmarking.

PIHH’s fault-tolerance validation focuses on the loss of functionally critical individual UAVs rather than mass simultaneous failure (30% to 50% swarm removal). In heterogeneous SEAD missions, removing all UAVs of a single functional type renders the reconnaissance→strike→assessment task chain physically incompletable, crossing from recoverable fault to mission failure. SoNS can evaluate high-loss-rate scenarios because its homogeneous or weakly heterogeneous swarms allow any survivor to assume any role. The 2 frameworks face different fault-tolerance boundaries, shaped by functional heterogeneity and task coupling.

## Materials and Methods

This section details the construction of the problem and algorithm. For the problem component (the “Problem description and instance construction” section), the “Problem description” section presents the problem description and mathematical model for the SEAD task, while the “Instance construction” section demonstrates how discrete event simulation systems are utilized to generate simulation instances. For the algorithm component (the “PIHH algorithm design” section), the “UAV/task/random strategy set” section defines the base strategy set, where interactions and combinations of base strategies generate advanced strategies. The “Two-layer network interaction structure based on self-attention mechanism” section provides the interaction structure governing strategy generation. The “Event-triggered joint selection meta-algorithm” section details the meta-algorithm responsible for deploying strategies to specific swarm tasks. The “Environmentally driven multiscale evolutionary approach” section outlines the training process for PIHH. Finally, the “PIHH and comparison algorithm settings” section presents the settings for PIHH and all comparison algorithms.

### Problem description and instance construction

#### Problem description

SEAD is a tactical doctrine that employs UAVs to neutralize air defense systems. It has demonstrated critical effectiveness in recent conflicts such as the Russia–Ukraine war and the Nagorno–Karabakh conflict. Specifically, SEAD constitutes a task chain comprising search, localization, reconnaissance, strike, and assessment phases. Previous research has predominantly focused on individual, isolated tasks within this chain [48]. However, in actual combat scenarios, numerous targets often require the execution of multiple, heterogeneous tasks with strict temporal constraints. For instance, reconnaissance, strike, and assessment typically need to be performed sequentially; such tasks are categorized as coupling tasks. Extensive studies have been conducted on coupling tasks, including 2-phase variants like search–strike [49] and search–rescue [50], as well as more complex 3-phase sequences such as reconnaissance–strike–assess [51]. Literature [52] indicates that the complexity of coupling tasks arises not only from temporal dependencies between tasks but also from the functional heterogeneity of the UAV swarm, i.e., systems composed of UAVs with diverse capabilities. Functionally heterogeneous swarms need to satisfy multiple functional constraints and spatiotemporal limitations. If the solution violates the constraints, it is deemed infeasible. Therefore, conventional self-organization methods struggle to achieve efficient cooperation.

The problem is formulated under the following assumptions: the operational environment is modeled as a 2-dimensional plane, neglecting terrain obstacles. All necessary information for task execution is assumed to be available beforehand, though this information may be subject to change. Both targets and UAVs are treated as point objects, neglecting collision volumes. UAVs possess circular fields of view and operational ranges; they can execute tasks upon approaching within a certain proximity to a target. Kinematic constraints of the UAVs are disregarded. UAVs have limited maximum task resources; a UAV exits the task environment once its resources are depleted. All UAVs depart from a known origin point. A UAV is considered to have exited the environment either upon depleting its task resources or upon reaching its maximum operational range.

Based on the above assumptions, the problem is described as follows: Within a known 2-dimensional area, there are *M* randomly distributed targets. Each target requires the execution of multiple tasks with sequential dependencies. Task attributes, such as location and resource requirements, are randomly generated. A fleet of *N* UAVs, equipped with different devices, departs from a known origin. They must complete as many tasks as possible within limited time, travel distance, and task resource constraints. Task execution by UAVs is subject to time window and type restrictions; improper allocation will lead to inefficiency or invalidation. UAV attributes, such as carried resource quantity, movement speed, and resource consumption rate, are also randomly generated. The ultimate objective is to maximize overall benefit through rational task-UAV allocation under fixed cost constraints. The main parameters and their descriptions for the problem model are listed in Table 7.

**Table 7.** Main parameter symbols of the model

Notation	Definition
Sets	
*T*	The set of targets, $T=\left\{T_{1},\;T_{2},\;\cdots T_{M}\right\}$, indexed by *j*
$T_{j}^{m}$	The set of tasks that need to be performed for the target *T_j_*, $T_{j}=\left\{T_{j}^{1},\;T_{j}^{2},\;\cdots ,\;T_{j}^{G_{j}}\right\}$, indexed by *m*
*U*	The set of UAVs, $U=\left\{U_{1},\;U_{2},\;\cdots ,\;U_{N}\right\}$, indexed by *i*
*R_i_*	The set of function types of UAVs
*S_i_*	The set of tasks to which UAV *U_i_* has been assigned
$I_{T_{j}^{m}}$	The set of UAVs that have performed tasks $T_{j}^{m}$
Parameters	
$p_{T_{j}}$	The coordinate of the target $T_{j}$ location
$v_{T_{j}^{m}}$	The value of task $T_{j}^{m}$
$b_{T_{j}^{m}}$	The resources required to accomplish task $T_{j}^{m}$
$r_{T_{j}^{m}}$	The type to which the task $T_{j}^{m}$ belongs
$d\left(\cdot ,\;\cdot \right)$	The Euclidean distance between 2 points
$d_{i}^{\mathrm{Max}}$	The maximum travel distance of the UAV *U_i_*
*P_i_*	The coordinates of the UAV *U_i_*
*B_i_*	The number of task resources the UAV *Ui* holds
$V_{i}$	The movement speed of the UAV *Ui*
*Q_i_*	The resource consumption rate of UAV *U_i_*
$C_{i,T_{j}^{m}}$	The resources consumed by *U_i_* for $T_{j}^{m}$
$C_{i}^{\mathrm{Max}}$	The resource limit by UAV *U_i_* for one task
${\mathrm{Num}}_{i,T_{j}^{m}}$	The times UAV *U_i_* performs task $T_{j}^{m}$
t	The current time
$t_{\max}$	The maximum running time
$t_{T_{j}^{m}}^{\text{start}}$	The time when task $T_{j}^{m}$ is first executed
$t_{T_{j}^{m}}^{\mathrm{end}}$	The time when task $T_{j}^{m}$ is completed
∗	The superscript indicating the specific entity (e.g., target, UAV, or task) that is impacted by the dynamic event
Decision variables	
$y_{T_{j}^{m}}$	Binary variable, whether the task $T_{j}^{m}$ has been completed
$x_{i,T_{j}^{m}}$	Binary variable, whether the task $T_{j}^{m}$ is being executed by UAV $U_{i}$
$\text{type}\left(R_{i},\;r_{T_{j}^{m}}\right)$	Binary variable, whether the types of task $T_{j}^{m}$ and UAV $U_{i}$ the same
$X_{i,T_{j}^{m}}$	Binary variable, whether, whether the task $T_{j}^{m}$ is allocated to UAV $U_{i}$

On the basis of defining the above symbols, we proceed to establish the mathematical model for the problem:

$$\mathrm{Maximize}\;Z=W_{1}Z_{1}-W_{2}Z_{2}-W_{3}Z_{3}$$ (1)

$$W_{1},W_{2},W_{3}\in {\mathbb{R}}^{+},Z_{1},Z_{2},Z_{3}>0$$ (2)

$$Z_{1}=\sum_{j\in \left\{1,\;2,\;\cdots ,\;M\right\}}\sum_{\;m\in \left\{1,\;2,\;\cdots ,\;G_{j}\right\}}\lambda_{T_{j}^{m}}v_{T_{j}^{m}}y_{T_{j}^{m}}$$ (3)

$$\begin{array}{c} \lambda_{T_{j}^{m}}\left(t\right)=\left\{\begin{array}{c} 0.5,t<E_{T_{j}^{m}}\\ \frac{t-E_{T_{j}^{m}}}{2\left(e_{T_{j}^{m}}-E_{T_{j}^{m}}\right)},E_{T_{j}^{m}}\leq t<e_{T_{j}^{m}}\\ 1,e_{T_{j}^{m}}\leq t<l_{T_{j}^{m}}\;\\ \frac{L_{T_{j}^{m}}-t}{2\left(L_{T_{j}^{m}}-l_{T_{j}^{m}}\right)},l_{T_{j}^{m}}<t\leq L_{T_{j}^{m}}\\ 0.5,t>L_{T_{j}^{m}} \end{array}\right. \end{array}$$ (4)

$$\begin{array}{c} Z_{2}=W_{4}\sum_{i\in \left\{1,\;2,\;\cdots ,\;N\right\}}\sum_{k\in S_{i}}d\left(P_{S_{i}^{k}},\;P_{S_{i}^{k+1}}\right)+W_{5}\sum_{i\in \left\{1,\;2,\;\cdots ,\;N\right\}}B_{i}+W_{6}N \end{array}$$ (5)

$$W_{4},W_{5},W_{6}\in {\mathbb{R}}^{+}$$ (6)

Subject to

$$\begin{array}{c} Z_{3}=\max_{j,m}t_{T_{j}^{m}}^{\mathrm{end}}y_{T_{j}^{m}},j\in \left\{1,\;2,\;\cdots ,\;M\right\},m\in \left\{1,\;2,\;\cdots ,\;G_{j}\right\} \end{array}$$ (7)

$$\begin{array}{c} \begin{array}{c} \sum_{j}\sum_{m}x_{i,T_{j}^{m}}\leq 1,i\in \left\{1,\;2,\;\cdots ,\;N\right\},j\in \left\{1,\;2,\;\cdots ,\;M\right\},m\in \left\{1,\;2,\;\cdots ,\;G_{j}\right\} \end{array} \end{array}$$ (8)

$$\begin{array}{c} 0\leq {\mathrm{Num}}_{i,T_{j}^{m}}\leq 1,i\in \left\{1,\;2,\;\cdots ,\;N\right\},j\in \left\{1,\;2,\;\cdots ,\;M\right\},m\in \left\{1,\;2,\;\cdots ,\;G_{j}\right\} \end{array}$$ (9)

$$\begin{array}{c} \begin{array}{c} \sum_{i}x_{i,T_{j}^{m}}\geq 0,i\in \left\{1,\;2,\;\cdots ,\;N\right\},j\in \left\{1,\;2,\;\cdots ,\;M\right\},m\in \left\{1,\;2,\;\cdots ,\;G_{j}\right\} \end{array} \end{array}$$ (10)

$$\begin{array}{c} \sum_{i}C_{i,T_{j}^{m}}\geq b_{T_{j}^{m}},i\in I_{T_{j}^{m}},j\in \left\{1,\;2,\;\cdots ,\;M\right\},m\in \left\{1,\;2,\;\cdots ,\;G_{j}\right\} \end{array}$$ (11)

$$\sum_{k}d\left(P_{S_{i}^{k}},\;P_{S_{i}^{k+1}}\right)<d_{i}^{\mathrm{Max}},i\in \left\{1,\;2,\;\cdots ,\;N\right\},k\in S_{i}$$ (12)

$$\begin{array}{c} \begin{array}{c} \sum_{j}\sum_{m}C_{i,T_{j}^{m}}X_{i,T_{j}^{m}}<B_{i},i\in \left\{1,\;2,\;\cdots ,\;N\right\},j\in \left\{1,\;2,\;\cdots ,\;M\right\},m\in \left\{1,\;2,\;\cdots ,\;G_{j}\right\} \end{array} \end{array}$$ (13)

$$\begin{array}{c} \begin{array}{c} C_{i,T_{j}^{m}}<C_{i}^{\mathrm{Max}},i\in \left\{1,\;2,\;\cdots ,\;N\right\},j\in \left\{1,\;2,\;\cdots ,\;M\right\},m\in \left\{1,\;2,\;\cdots ,\;G_{j}\right\} \end{array} \end{array}$$ (14)

$$\begin{array}{c} t_{T_{j}^{m}}^{\mathrm{end}}<t_{T_{j}^{m}}^{\text{start}},j\in \left\{1,\;2,\;\cdots ,\;M\right\},m\in \left\{1,\;2,\;\cdots ,\;G_{j}\right\} \end{array}$$ (15)

$$D=\left\{d_{1},\;d_{2},\;d_{3}\right\}$$ (16)

$$d_{1}:T\leftarrow T\cup T^{*},T^{*}\notin T$$ (17)

$$d_{2}:\exists T_{j}^{m}\in T,T_{j}^{m}\leftarrow T_{j}^{m_{*}},m_{*}\neq m$$ (18)

$$d_{3}:U\leftarrow U\backslash U^{*},U^{*}\in U$$ (19)

Equations (1) to (7) constitute the objective function of the model. Equation (1) represents the overall objective function Z, composed of 3 components: task benefit *Z*_1_, UAV utilization cost *Z*_2_, and time cost *Z*_3_. In Eq. (2), $W_{1},W_{2},W_{3}$ denote the respective weights of the 3 objectives. In Eq. (3), task payoff *Z*_1_ represents the reward obtained after the UAV completes the task. Equation (4) is the time window function $\lambda_{T_{j}^{m}}\left(t\right)$, indicating the quality of the execution timing *t* for task $T_{j}^{m}$. $\left[E_{T_{j}^{m}},\;e_{T_{j}^{m}}\right)$ represents the rising phase of the time window function; $\left(e_{T_{j}^{m}},\;l_{T_{j}^{m}}\right)$ represents the peak phase; $\left(l_{T_{j}^{m}},\;L_{T_{j}^{m}}\right]$ represents the falling phase; task execution outside the time window $\left[E_{T_{j}^{m}},\;L_{T_{j}^{m}}\right]$ is considered invalid. Equation (5) is the UAV usage cost *Z*_2_, composed of 2 parts. The UAV consumes energy during movement, with energy costs related to travel distance and the weight of resources carried. Additionally, fixed costs exist for UAV operation, such as equipment purchase and maintenance. In Eq. (6), $W_{4},W_{5}$ represent the weights for travel distance and resource weight, respectively; $W_{6}$ denotes the fixed cost per UAV. Equation (7) is the time cost *Z*_3_, recording the time all UAVs take to complete the final task. When all tasks are completed, time cost *Z*_3_ aids in evaluating the overall system efficiency. A shorter completion time for the final task indicates efficient execution before resource depletion, while a longer time suggests either suboptimal task allocation or failure to utilize resources at appropriate moments.

Equations (8) to (19) constitute the constraints, comprising 4 components: synchronization constraints, resource constraints, timing constraints, and uncertainty sets. Specifically, Eqs. (8) to (10) represent synchronization constraints, which define the conditions UAVs must adhere to when cooperating to complete tasks. Specifically, Eq. (8) restricts UAVs to executing only a single task per time step, effectively preventing resource contention caused by concurrent multitasking. Equation (9) prohibits UAVs from repeatedly executing the same task, preventing resource waste from redundant operations and potential revenue loss due to missed windows. Equation (10) defines the parallel task execution mechanism for multiple UAVs, allowing several UAVs to collaboratively execute a single task within the same time step by jointly consuming resources. Equations (11) to (14) represent resource constraints quantifying the boundary conditions for UAV task allocation. Specifically, Eq. (11) indicates that when multiple UAVs collaborate on a task, the task is considered completed if the total resources consumed by all UAVs equals the task’s resource requirement, thereby preventing resource waste from redundant collaboration or task failure due to resource shortages. Equation (12) imposes a maximum movement distance constraint on UAVs to avoid frequent long-distance task adjustments. Equation (13) indicates that the total resources consumed by a UAV for its assigned tasks must be less than the resources it carries. Equation (14) represents a limit on the total resources consumed by a UAV for a single task, reducing extreme cases of resource shortages or overloading. Equation (15) is a temporal constraint reflecting the multistage coupling characteristics of tasks, where each task must begin only after the preceding one is completed. Equations (16) to (19) constitute an uncertainty set denoted by *D*. This set encompasses unexpected events across 3 levels: environmental, task, and UAV. Environmental-level events include the emergence of previously undetected new targets (*d*_1_); task level events involve errors in task execution status (*d*_2_); and UAV-level events encompass irreversible functional failures or malicious damage (*d*_3_). The asterisk (*) in Eqs. (16) to (19) is a superscript indicating the specific entity (e.g., target, UAV, or task) that is impacted by the dynamic event.

In summary, the preceding discussion establishes a problem model and enumerates emergency scenarios. It is evident that this constitutes a complex optimization problem characterized by severe resource constraints and spatiotemporal limitations, where achieving cooperative behavior under static conditions is already highly challenging. However, the randomness inherent in the type, timing, and location of emergencies further drastically reduces the number of viable cooperative solutions. Notably, PIHH is a model-free algorithm that can adapt its strategy based on environmental feedback, thereby overcoming the limitations of passive, fixed approaches.

#### Instance construction

**Fig. 11.**
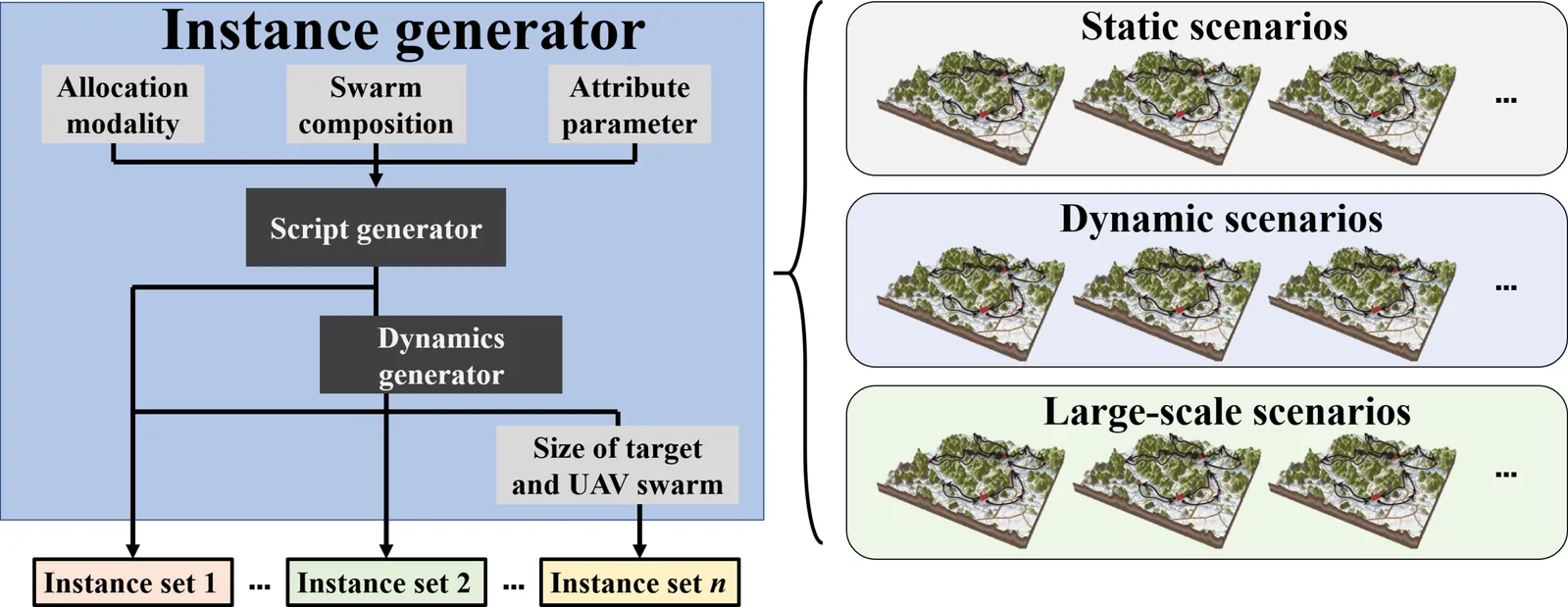
Scene composition and instance generator.

The generation of scenario instances employs the discrete event simulation system depicted in Fig. 11. This simulation system utilizes a parametric modeling approach, wherein a scenario comprises static factors (including allocation patterns, swarm composition, task attribute sets, and UAV attribute sets) and dynamic factors (including type, time, location, and severity). Specifically:

• Allocation pattern. Since each target requires 3 types of tasks to be executed, the actual number of tasks is 3*M*. The relationship between target *M* and UAV *N* is *N* = 0.5*M*. Research indicates that among all combinations of *M* and *N*, the scenario where *N* < *M* is the most complex. The challenge lies in how to utilize fewer UAVs to accomplish complex tasks while managing temporal relationships.
• Swarm composition. The ratio of reconnaissance UAVs, strike UAVs, and integrated UAVs is set at 0.4/0.4/0.2. Reconnaissance and strike UAVs possess entirely different functionalities, requiring complementary cooperation to complete tasks. Integrated UAVs can execute all tasks but are deployed in limited numbers, serving to ensure a baseline task capability during high-incidence scenarios.
• Task attribute set. Tasks are defined as quadruplets: <Location, Value, Resources, Type>. Location is a random value within the range [(0, 0), (10, 10)]; Value is 2; Resources are 2; Type can be reconnaissance, strike, or assessment. Upon completion, Reconnaissance and Strike tasks transition to the next type. Upon completion of an Assessment task, the target exits the environment.
• UAV attribute set. UAV attributes can be represented as a 6-tuple: <Position, Ability, Resources, Speed, Radius, Type>. Initial position is (0, 0); Ability is a random value within [0.5, 1]; Resources are 30; Speed is a random value within [1.5, 2]; Radius is 0.25, and only tasks within this radius can be executed; Types include reconnaissance, strike, and integrated. Reconnaissance UAVs can perform reconnaissance and assessment tasks; strike UAVs can perform strike tasks; integrated UAVs can perform all tasks.
• Dynamic factors. Dynamic factors include new target emergence, UAV failure, and task information error. Their occurrence types are set as single occurrence, pairwise combination, and random type. The occurrence rate of dynamic factors is denoted as *P*(*D*), representing the ratio of unexpected events to the total number of targets. For single occurrence and pairwise combinations, the dynamic factor type is predetermined. For random types, the dynamic factor type is randomly selected. Once the preset upper limit *P*(*D*) is reached, no further dynamic factors are generated. Regardless of the type setting, the position of dynamic factors is a random value within the range [(0, 0), (10, 10)], and the time is a random value before all tasks are completed. This ensures that each generated scenario is entirely distinct.

In summary, the discrete-event simulation system can generate a large number of completely distinct scenario instances. This capability facilitates testing under highly dynamic and disruptive environmental conditions, as well as with ultra-large-scale swarms that are challenging to deploy in physical reality. Each scenario in the experiment was independently and randomly generated 30 times to minimize subjectivity. The experimental environment ran on the MATLAB R2017 software platform, equipped with an Intel Core i7-12700 processor operating at a clock speed of 2.10 GHz.

### PIHH algorithm design

#### UAV/task/random strategy set

Base strategies represent irreducible rules extracted from problem characteristics. Each base strategy possesses independent functional semantics, such as task distance and resource matching. Advanced strategies are formed through the interaction and combination of multiple base strategies. Because of high problem dependency, strategy sets across different problems are often difficult to directly interchange. Therefore, it is necessary to design dedicated strategy sets tailored to the SEAD problem. We propose 3 types of strategy sets, each incorporating base strategies that address distinct aspects of the SEAD problem: UAV-based, task-based, and random-based strategies. The base strategy set comprises 18 base strategies in total, as shown in Table 8. Furthermore, considering the highly limited onboard resources of common UAV platforms, computational efficiency is a key design principle for this base strategy set. As illustrated in Table 8, all operations within the base strategy set exhibit computational complexity of O(1), O(*N*), or O(*M*) for simple computations.

**Table 8.** Base strategy set

Strategy	Description	Complexity	Type
Strategy 1	Distance of the UAV from other companions	O(*N*)	UAV
Strategy 2	Distance of the UAV from the current task	O(1)	
Strategy 3	Distance of the UAV from all functional compliance tasks	O(*M*)	
Strategy 4	Number of tasks completed by the UAVs	O(1)	
Strategy 5	Number of functions of the UAVs	O(1)	
Strategy 6	Percentage of UAVs with the same functionality as the current UAV in the swarms	O(*N*)	
Strategy 7	Number of tasks that UAVs can perform as a percentage of all tasks	O(*M*)	
Strategy 8	Number of remaining resources for this UAV	O(1)	
Strategy 9	Movement speed of the UAV	O(1)	
Strategy 10	Resource consumption rate of the UAV	O(1)	
Strategy 11	Distance of the task from other tasks	O(*M*)	Task
Strategy 12	Distance of the task from all functionally compatible UAVs	O(*N*)	
Strategy 13	Percentage of UAVs in the swarm that are functionally aligned with the task	O(*N*)	
Strategy 14	The value of the time window function for the task	O(*N*)	
Strategy 15	Number of resources required for the task to be completed	O(1)	
Strategy 16	Order of execution of the task	O(1)	
Strategy 17	The heuristic value computed in the previous round	O(1)	Random
Strategy 18	Random constants between [0,1]	O(1)	

The selection of the 18 base strategies listed in Table 6 is grounded in a systematic design philosophy that prioritizes coverage of essential decision factors, computational efficiency, and semantic interpretability, while also ensuring the set is both complete and minimally redundant. To achieve comprehensive coverage, the SEAD problem is decomposed into 3 orthogonal dimensions—UAV status, task characteristics, and exploratory behavior—each addressed by a dedicated subset of strategies. UAV-based strategies (Strategies 1 to 10) capture individual states such as remaining resources, speed, and functional type, as well as relational attributes like distances to companions and to compatible tasks. Task-based strategies (Strategies 11 to 16) describe spatial distribution, functional requirements, temporal urgency, and execution order of the tasks. Random-based strategies (Strategies 17 and 18) introduce stochasticity and memory of past decisions to foster exploration and prevent premature convergence. Together, these 18 strategies span the spatial, temporal, functional, and exploratory facets that military domain experts often identify as critical for cooperative mission planning.

Computational efficiency is another cornerstone of the design: every strategy is formulated to be computable in constant or linear time with respect to the number of UAVs or tasks, ensuring that the HH can run online on resource-limited platforms without prohibitive delay.

Although the current strategy set is tailored to the SEAD context, its modular design readily generalizes to other robot-swarm tasks. It only requires a semantic reinterpretation. For example, in a USAD scenario, the concept of “task” is reinterpreted as a “survivor location” with associated urgency and required rescue resources. Accordingly, Strategy 14 (time-window function) becomes a victim survival probability window, while Strategy 12 (distance from task to functionally compatible UAVs) becomes the distance from the victim to capable rescue UAVs, and Strategy 15 (resources required for the task) becomes the medical supplies or equipment needed for the rescue. Most other strategies—such as distances (Strategies 1 to 3 and 11), UAV states (Strategies 4 and 8 to 10), functional proportions (Strategies 5 to 7 and 13), execution order (Strategy 16), and exploratory heuristics (Strategies 17 and 18)—are domain-independent and can be reused directly, as their underlying computations remain unchanged. Only the meaning of “function”, “resource”, and “task” is updated to match the new domain. New strategies, such as communication link quality, can also be added if the target problem introduces additional dimensions. In the “PIHH deployment to the USAD mission” section, we presented an adaptation method for USAD to demonstrate its portability.

**Fig. 12.**
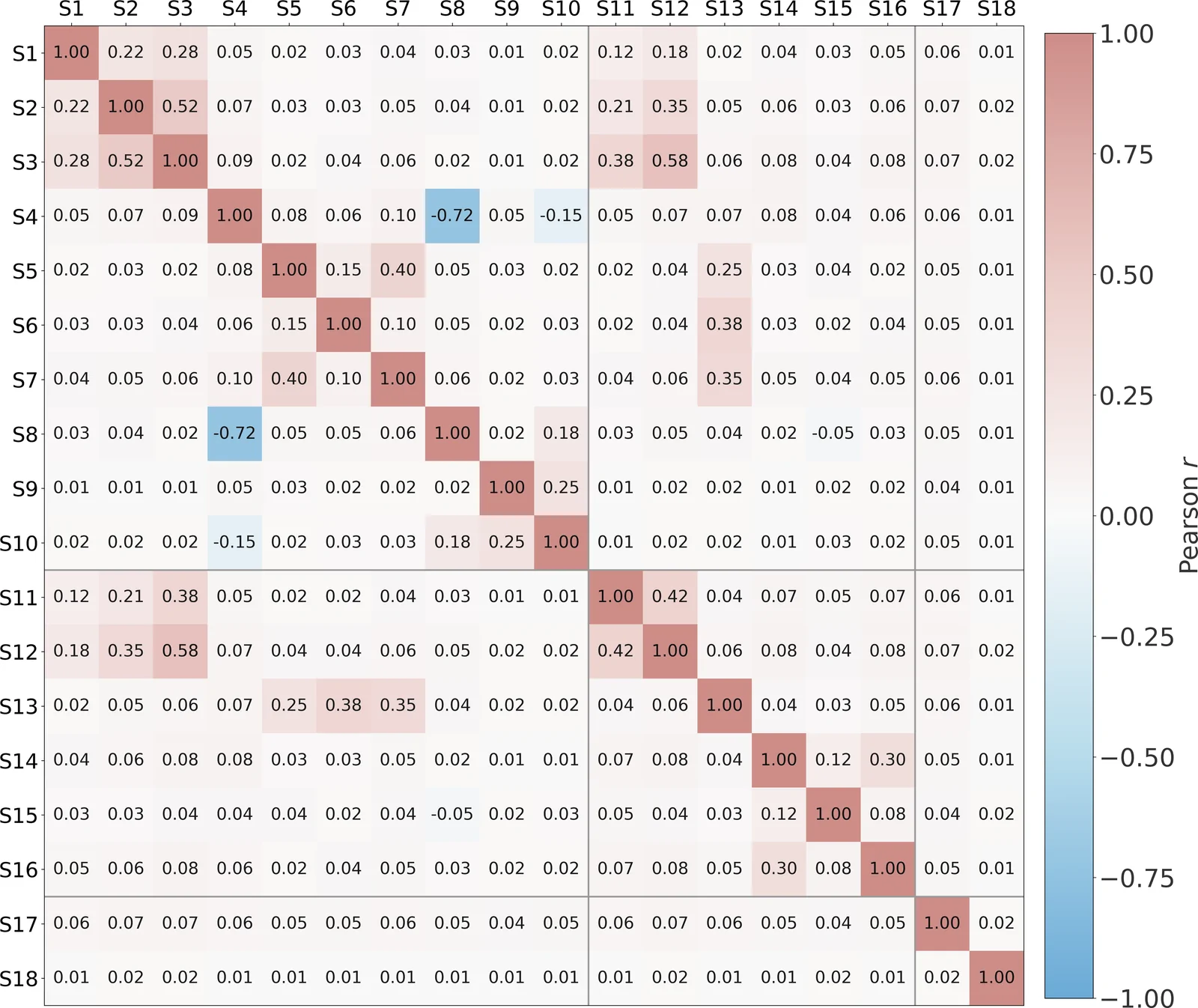
Pairwise Pearson correlation coefficient matrix of the 18 base strategies.

To quantitatively examine the minimally redundant design principle of the 18 base strategies listed in Table 8, we compute all 153 pairwise Pearson correlation coefficients across 500 randomly sampled environmental states, covering a factorial combination of *N* in {10, 50, 100, 250}, *M* in {50, 100, 200, 500}, and *P*(*D*) in {0.1, 0.2, 0.3, 0.5}. In each sampled state, all strategy values are extracted and normalized to the [0,1] interval. The complete numerical matrix is presented in Fig. 12.

Table 9 summarizes the pairs with |*r*| > 0.30 and their structural origins. The mean |*r*| across all 153 pairs is 0.08. The maximum |*r*| across all cross-category pairs (e.g., distance–resource, distance–function, and resource–temporal) is only 0.09.

**Table 9.** Strategy pairs with |*r*| > 0.30 and their structural origins

No.	Pair	*r*	Structural origin
1	S4–S8	−0.72	Complementary measures of resource consumption: more tasks completed means fewer remaining resources
2	S3–S12	0.58	Near-symmetric mathematical form: UAV to tasks vs. tasks to UAV
3	S2–S3	0.52	Shared UAV coordinates and task distribution; S2 perturbed by allocation decisions
4	S11–S12	0.42	Shared task spatial distribution
5	S5–S7	0.40	More function types lead to higher task-type coverage
6	S6–S13	0.38	Functional matching from UAV-side and task-side perspectives
7	S3–S11	0.38	Both are spatial set averages, from different reference frames
8	S2–S12	0.35	Shared UAV-task spatial distribution
9	S7–S13	0.35	Bidirectional measures of functional coverage
10	S14–S16	0.30	Tighter time windows drive higher execution priority

The matrix exhibits 3 structural features. First, moderate correlations (0.35 to 0.58) cluster among distance-based strategies (S1 to S3, S11, and S12), reflecting shared spatial variables; differences in reference frame and aggregation level keep all pairs below the collinearity threshold of 0.85. Second, the strong negative correlation between S4 and S8 (−0.72) follows from the resource–consumption constraint: each completed task consumes a fixed amount of resources. The 2 strategies characterize a UAV’s resource state from complementary perspectives. Third, cross-category correlations are near zero, consistent with the 3 orthogonal design dimensions in Table 8 (UAV, task, and random).

Self-attention weights are determined by Query–Key matching followed by Softmax normalization, which is equivariant to linear scaling of input features [53]. When 2 strategies are moderately correlated, Query vectors encoding current environmental demands can assign higher weight to the more decision-relevant strategy. The differentiated activation frequencies in Fig. 5 (Strategy 11: ~4,700 vs. Strategy 2: ~3,500) confirm that the self-attention mechanism distinguishes between correlated but functionally distinct inputs. Moderate correlations among distance strategies do not cause weight dilution or over-concentration.

#### Two-layer network interaction structure based on self-attention mechanism

Based on the aforementioned strategy set as base components, PIHH aims to generate new algorithms for problem-solving. Consequently, the design of the new algorithm generation mechanism becomes pivotal in determining PIHH’s performance. Fission–fusion behavior in primates provides inspiration for this problem. Reference [12] articulates primate interaction mechanisms through 3 key questions: where interactions occur, who selects interaction partners, and what constitutes the context for interactions. Primates employ a goal-oriented mechanism for selecting interaction partners, enabling individuals to construct social niches across diverse contexts and thereby optimize their behavior. Inspired by this process, the design of the interaction structure must satisfy the following 2 requirements: Requirement 1 (each strategy must be able to interact with all other strategies) and Requirement 2 (the order of strategies within the set should not influence interaction outcomes).

To address Requirement 1, this paper designs a 2-layer network strategy interaction structure. This 2-layer network interaction simulates the fission–fusion societies of primates. Strategies cyclically interact between the 2 layers, forming temporary subpopulations during interactions. This allows strategies to undergo local reorganization and optimization before gradually propagating to the whole. The strategy interaction structure comprises 2 strategy layers with identical compositions. The outcome of strategy interaction is the gradual generation of higher-order strategies not present in the base strategy set.

To address Requirement 2, the specific computation of strategy interaction employs a self-attention mechanism. This mechanism exhibits permutation equivariance, meaning an individual’s output depends on its relationship with the entire population, not its order within it. The self-attention mechanism dynamically calculates the similarity between the demand features (Query) and the matching features (Key) of strategies. This simulates the process by which primates select interaction partners through direct learning and indirect associations. It enables autonomous attention to other strategies that have demonstrated high returns historically and facilitates the adjustment of one’s own behavior.

The feature mapping of the self-attention mechanism contains 3 components: the base input (Value), the demand feature (Query), and the matchable feature (Key). In this paper, the normalized value *Va* of each strategy is used as the base input. The normalized value is computed using min–max normalization:

$${\mathrm{Va}}_{s}=\frac{V_{s}-V_{\min}}{V_{\max}-V_{\min}},s\in \left\{1,\;\cdots ,\;18\right\}$$ (20)

where *V_s_* is the raw value of strategy *s* (e.g., distance and resource count), and *V*_min_ and *V*_max_ are its theoretical or empirical bounds. For strategies without natural bounds, empirical bounds are collected from historical task data.

The demand feature matrix $w_{Q}\in {\mathbb{R}}^{18\times d}$ and matching feature matrix $w_{K}\in {\mathbb{R}}^{18\times d}$ are both set with dimension *d* = 18, equal to the number of base strategies. This ensures that each strategy is represented by a dedicated feature vector of length 18, enabling full pairwise interaction modeling.

For a given interaction round, strategies are organized into 2 layers: Layer 1 and Layer 2, each containing the same set of 18 strategies. Let ${\mathrm{Va}}^{\left(1\right)}$ and ${\mathrm{Va}}^{\left(2\right)}$ denote the normalized value vectors of the 2 layers. The demand feature for strategy *x* in Layer 1 is computed as:

$$Q_{x}=w_{Q}^{Τ}\cdot {\mathrm{Va}}_{x}^{\left(1\right)},$$ (21)

and the matching feature for strategy *y* in Layer 2 is:

$$K_{y}=w_{K}^{Τ}\cdot {\mathrm{Va}}_{y}^{\left(2\right)}.$$ (22)

The self-attention weight from strategy *x* (Layer 1) to strategy *y* (Layer 2) is given by:

$$a_{\mathrm{xy}}=\frac{\exp\left(\frac{Q_{x}\cdot K_{y}}{\sqrt{d}}\right)}{\sum_{z=1}^{18}\exp\left(\frac{Q_{x}\cdot K_{z}}{\sqrt{d}}\right)}.$$ (23)

The heuristic value for strategy *x* is then obtained by aggregating information from all strategies in Layer 2:

$$h_{x}=\sum_{y=1}^{18}a_{\mathrm{xy}}\cdot {\mathrm{Va}}_{y}^{\left(2\right)}.$$ (24)

The strategy with the highest heuristic value in Layer 1 is selected as the winner, and its index and heuristic value are recorded. After each interaction, the demand and matching feature matrices are updated using a neighborhood function and learning rate, as described in Algorithm 3. The 2 layers alternate roles in successive interactions, enabling bidirectional information flow and continuous strategy evolution.


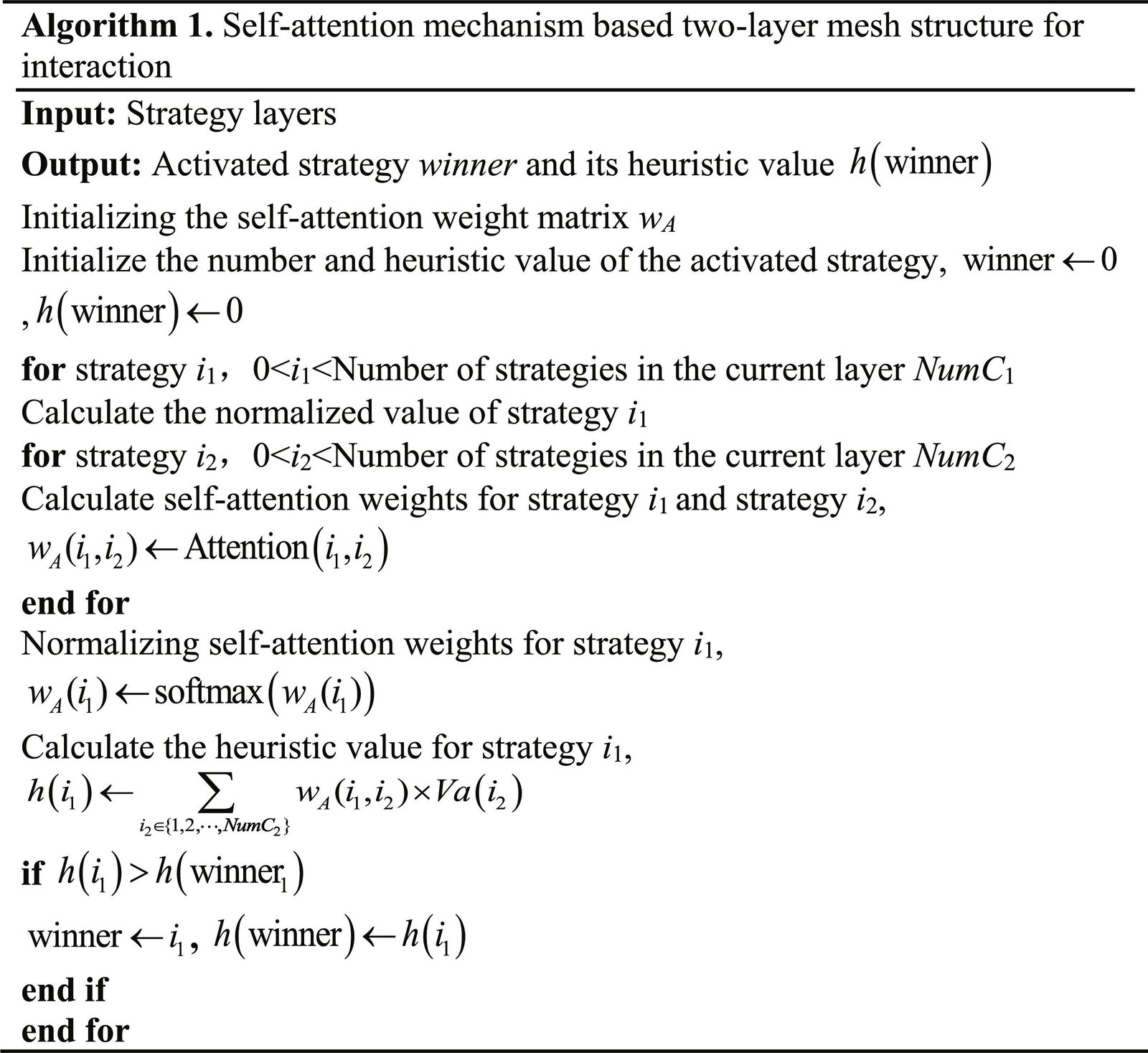


Algorithm 1 gives a single strategy interaction process based on the self-attention mechanism. In a given interaction, assume that the strategy layer in which the activation takes place is strategy layer 1. The strategies in strategy layer 1 are traversed so that self-attention weights are computed with each strategy in strategy layer 2. The information of all strategies in strategy layer 2 is aggregated based on the self-attention weights, which is recorded as the heuristic value. Unlike a global fitness value, the heuristic value aggregates local metrics to evaluate the current suitability of a specific UAV for a specific task. Finally, the strategy with the largest heuristic value in strategy layer 1 is selected as the activated strategy. The number and heuristic value of the activated strategy are recorded. Subsequently, the next interaction is carried out, and the activated strategy layer is strategy layer 2. The above process is executed cyclically until the maximum number of interactions is reached. In summary, the strategies in the ensemble use a self-attention mechanism to perform a 2-layer network-like self-organized interaction. This process promotes the co-evolution of effective swarm policies among embodied UAVs through information interaction.

This dual-layer design parallels the spatiotemporal decomposition in attention-based spatio-temporal graph neural network (AST-GNN) [54], which decouples multiagent interaction into alternating spatial and temporal Transformer modules. In CE&SG, cross-strategy weight aggregation handles the spatial dimension, and the cyclic alternation between the 2 strategy layers handles the temporal dimension, both within a unified self-attention mechanism.

Self-attention in Algorithm 1 operates at the strategy level: attention weights are computed between the 18 base strategies via Eqs. (21) to (23), satisfying Requirement 1 in the “Two-layer network interaction structure based on self-attention mechanism” section that every strategy can interact with every other strategy. This does not imply a fully connected communication topology among robots. During deployment, each robot computes strategy feature vectors from local sensor data and exchanges them only within its subgroup. Subgroups form through cosine similarity clustering, and their sizes are governed by fission–fusion dynamics. Communication scope is determined by subgroup size, not by *N*.

PIHH uses “weight” in 2 senses. Self-attention parameter matrices (*w_Q_*, *w_K_*, and *w_V_*) are learned offline and fixed during deployment. Strategy composition weights (Softmax attention scores) are computed online from the current environmental state at each decision cycle. The experimental validation covers *N* ∈ {10, …, 250}, *M* ∈ {50, …, 500}, and *P*(*D*) ∈ {0.1, …, 0.5}. Strategy activation differentiation across these conditions (Fig. 5) and weight reorganization after dynamic events (see the “Case study: Patterns of strategy evolution in SEAD” section) have been verified within this range. Out-of-distribution generalization testing remains future work.

#### Event-triggered joint selection meta-algorithm

Meta-algorithm deploys strategy to specific swarm collaboration tasks. Meta-algorithm’s workflow starts with a null task allocation scheme, which is incrementally scaled up through incremental scaling from the end of the UAV task queue. Specifically, meta-algorithm obtains real-time, online environmental data from UAV-mounted sensors through an environmental situational awareness module. When a UAV completes the current task and has remaining resources, the meta-algorithm is triggered, and it dynamically selects and assigns appropriate subsequent tasks based on real-time data analysis from the environmental situational awareness module. This mechanism shifts the key decision-making aspect of task allocation from the preplanning stage to the execution stage, enabling the algorithm to make dynamic adjustments based on real-time environmental information. It has been shown that meta-algorithm exhibits excellent efficiency advantages in solving many decision problems, and has the potential to locally satisfy the real-time requirements of dynamic assignment scenarios.

The meta-algorithm (Algorithm 2) operates online and deploys strategies to specific swarm collaboration tasks. It sequentially performs task selection and UAV selection, each of which repeatedly invokes the self-attention mechanism (Algorithm 1) for forward computation using the fixed, offline-trained weight matrices. No training occur during online execution.


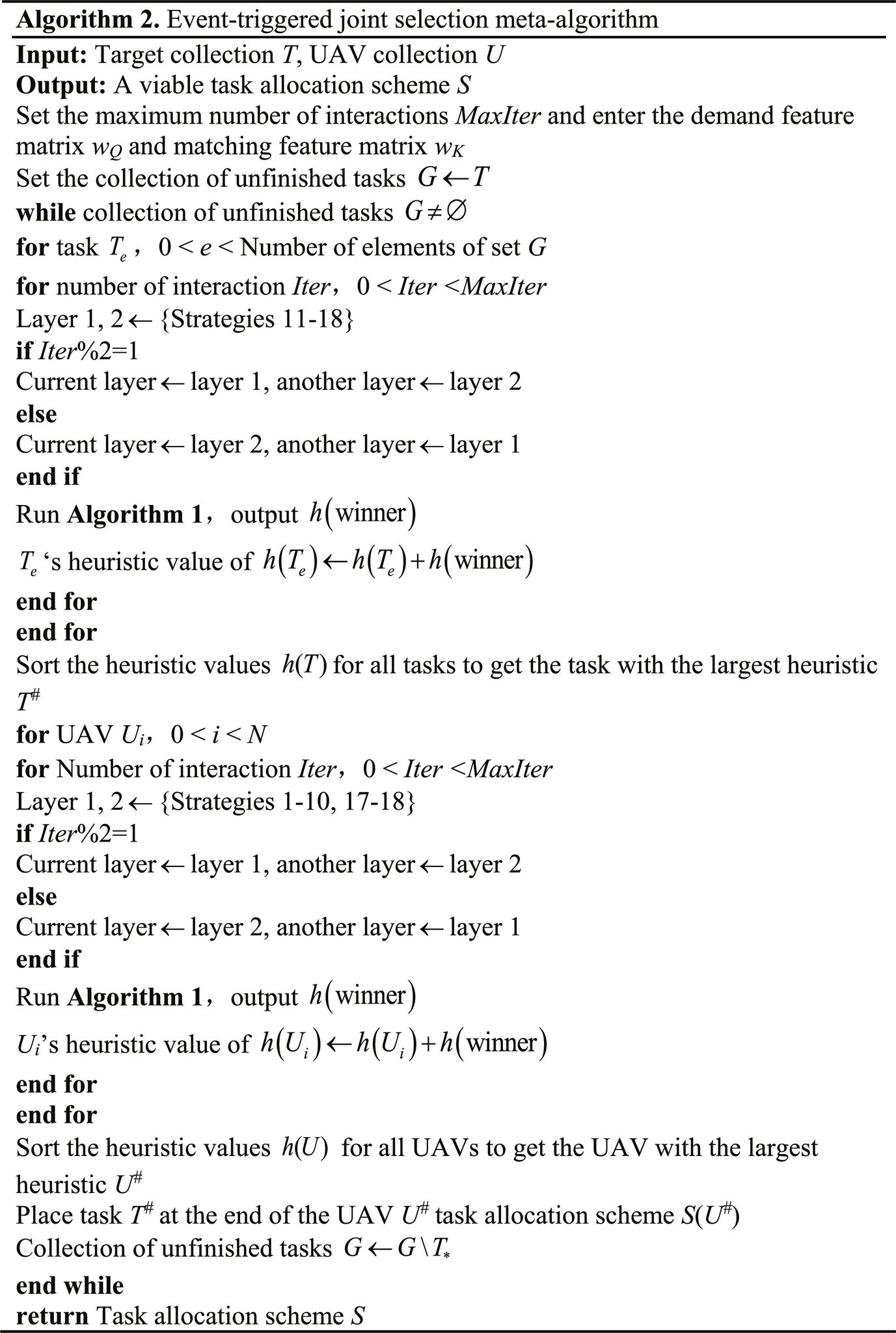


However, existing common meta-algorithm approaches typically only dynamically select UAVs, while task selection is performed by randomly choosing from the set of uncompleted tasks [42,55]. When addressing SEAD problems involving time-window benefits and temporal constraints, this common meta-algorithm exhibits blindness, potentially leading to missed task windows or ineffective allocations. To address these limitations, this paper proposes a joint selection of tasks and UAVs using meta-algorithm. Specifically, during the task selection phase, the 2 strategy layers within the strategy interaction structure comprise a task-based strategy set and a random strategy set, respectively. During the UAV selection phase, the 2 strategy layers within the strategy interaction structure comprise a UAV-based strategy set and a random strategy set, respectively. In summary, meta-algorithm triggers operation and adjusts strategy based on real-time data from physical interactions. The improved meta-algorithm in this paper enables situational awareness and joint selection for both task and UAV information, thereby further enhancing task assignment efficiency. The complete steps are detailed in Algorithm 2.

#### Environmentally driven multiscale evolutionary approach

To enable the algorithm to be generalizable in variable environments, the training process of PIHH is conducted in multiple types of environments, leading to multiscale evolution of its capabilities. The training objectives are set to enhance the adaptability to emergent environments and scalability to UAV/task scale expansion. In addition, although the static environment is not the main application scenario for the PIHH, it is still taken into account as an important metric. The algorithms designed by PIHH are expected to have the following properties: (a) maintain fast recovery under dynamic disturbances; and (b) maintain performance stability when the problem scale scales up.

The experimental design utilizes an evaluation system for multiple types of environments: static environments (E1), dynamic interference environments (E2), and large-scale problems (E3). The algorithm synthesis performance score (SR) is calculated by the weighted ranking method:

$$\mathrm{SR}=w_{E1}\times {\text{Rank}}_{E1}+w_{E2}\times {\text{Rank}}_{E2}+w_{E3}\times {\text{Rank}}_{E3}$$ (25)

where the weight coefficients reflect the degree of emphasis in different environments, $w_{E1}=0.2$, $w_{E2}=0.4$, $w_{E3}=0.4$; ${\text{Rank}}_{E1}$ denotes the performance ranking of the algorithm in static environment; ${\text{Rank}}_{E2}$ and ${\text{Rank}}_{E3}$ denote the performance ranking of the algorithm in dynamic environment and large-scale problems, respectively. The evaluation mechanism can be flexibly adapted to the prioritization requirements of different application scenarios through parameter adjustment.

Algorithm 3 gives the complete flow of a single algorithm training session. By periodically switching between different training environments, the algorithm is encouraged to develop greater generalization capabilities. Detailed information on the training environments can be found in the “PIHH and comparison algorithm settings” section.


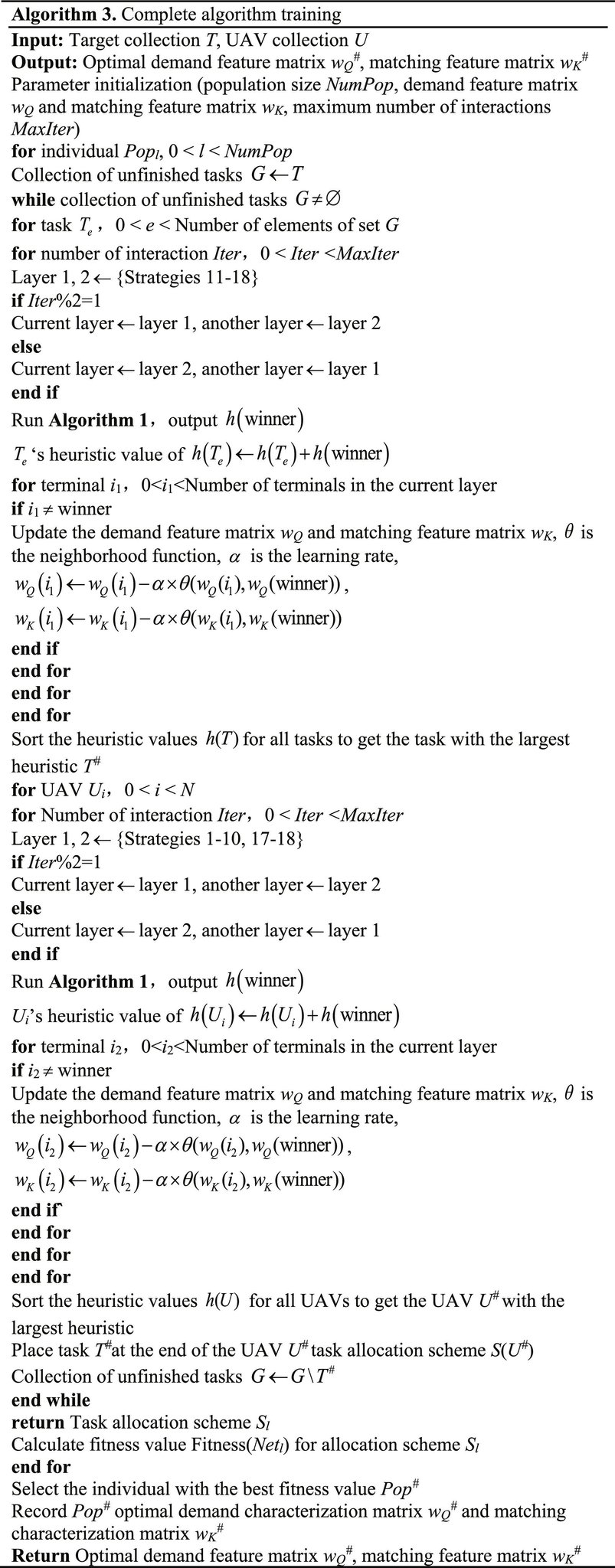


#### PIHH and comparison algorithm settings

The population size NumPop of PIHH is 100, the maximum number of iterations MaxIter is 50, the neighborhood function θ is a Gaussian function, and the learning rate is 0.5. Subsequent parameter experiments demonstrated the validity of these settings. Furthermore, PIHH does not require function operators, unlike common GPHH algorithms (which typically rely on {+, −, ×, /} operators, Boolean operators, and functions such as {min, max, sin, cos, log, …} for complex computations), resulting in lower computational overhead.

The population size for CMA-ES is set to 100, with 250 iterations. To accommodate dynamic problems, a partial restart mechanism is integrated into CMA-ES, whereby UAVs affected by sudden events must return to the initial point for reallocation. DCM parameters are configured as specified in the original text. The population size for GPHH is set to 100, with 50 iterations. The minimum and maximum tree depths are set to 2 and 6, respectively. GPHH-EM employs an elite size of 0.08 times the population size based on GPHH’s base. To ensure fair comparison, GPHH and GPHH-EM utilize the same training environment as PIHH. All algorithms aim to maximize the fitness function value. Additionally, the HH algorithm requires pretraining. We employ the same multitype environment for PIHH, GPHH, and GPHH-EM. The training environment consists of 3 base scenario instances: a small-scale static problem [*N* = 5, *M* = 10, *P*(*D*) = 0], a small-scale dynamic problem [*N* = 5, *M* = 10, *P*(*D*) = 0.3], and a large-scale static problem [*N* = 25, *M* = 50, *P*(*D*) = 0]. The optimal strategy from these is selected for validation.

It is worth noting that compared to the validation environment, the scale and number of agents in the 3 training environments are significantly smaller. This design stems from the fact that real-world environments are typically far more complex than virtual ones. Therefore, training does not involve setting fixed parameters in one step but rather provides a foundation for adaptability and scalability, enabling practical and continuous adjustments in subsequent unknown environments. Experimental results demonstrate that PIHH can generate effective response strategies even when confronted with more complex and unknown environments.

#### PIHH deployment to the USAD mission

**Fig. 13.**
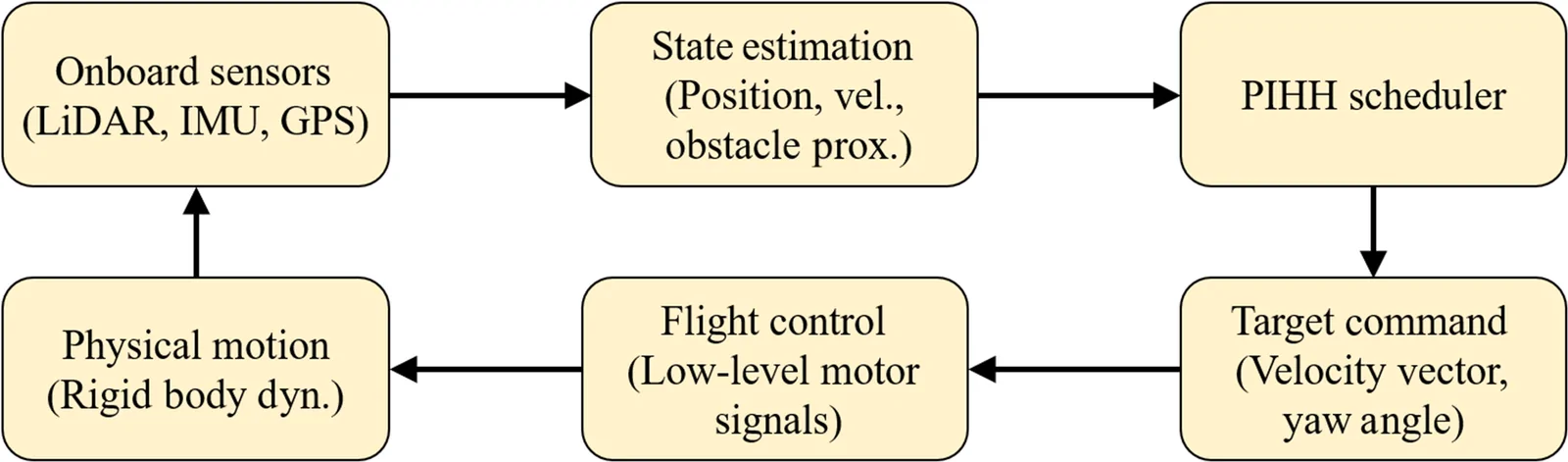
Integration architecture of PIHH for the USAD mission in AirSim.

Figure 13 gives the integration architecture of PIHH for the USAD mission in AirSim. The flowchart comprises 5 sequential modules connected by directed arrows, forming a closed control loop. Module descriptions:

• Onboard sensors: Light Detection and Ranging provides point cloud data for obstacle detection and relative positioning; IMU supplies acceleration and angular velocity; GPS offers coarse global localization.
• State estimation: Fuses sensor data to estimate each UAV’s current pose, velocity vector, and proximity to static obstacles and neighboring UAVs. This module outputs a structured state vector consumed by the PIHH scheduler.
• PIHH scheduler: The core decision-making module implementing the CE&SG framework. It ingests the real-time state vector, executes the event-triggered meta-algorithm (Algorithm 2), and invokes the self-attention mechanism (Algorithm 1) to generate a dynamically updated task allocation scheme. Crucially, the heuristic computation now incorporates a collision-risk metric derived from relative velocities and separation distances.
• Target command: The scheduler outputs a target velocity vector and yaw angle command for each UAV, translating the abstract task assignment (e.g., “proceed to survivor S3”) into a concrete motion directive.
• Flight control: The onboard low-level controller converts the target velocity and yaw commands into motor actuation signals, respecting the UAV’s dynamic constraints.
• Physical motion: AirSim’s physics engine simulates rigid-body dynamics, aerodynamic effects, and collision responses.

The transition from the discrete-event simulation (SEAD) to the continuous physics simulation (USAD) required 2 principal modifications to the PIHH framework: semantic reinterpretation of the base strategy set and integration of kinematic constraints.

1. Semantic reinterpretation of base strategies

The CE&SG architecture decouples the strategy interaction mechanism from the problem-specific semantics of base strategies. Consequently, migrating PIHH from the SEAD domain to the USAD domain does not require any alteration to the core algorithmic logic (Algorithms 1 to 3); only the interpretation of the 18 base strategies is updated to reflect the new task context. Table 10 presents the semantic mapping between the 2 scenarios, with all mathematical computations remaining identical to ensure algorithmic consistency and transferability.

**Table 10.** Semantic mapping of base strategies in SEAD and USAD scenarios

No.	SEAD	USAD	Computation logic changed?
Strategy 1	Distance between UAV and other companions	Distance between UAV and other companions	No
Strategy 2	Distance between UAV and current task	Distance between UAV and current survivor	No
Strategy 3	Distance between UAV and all functionally compatible tasks	Distance between UAV and all survivors with executable tasks	No
Strategy 4	Number of tasks completed by the UAV	Number of tasks (scan/delivery) completed by the UAV	No
Strategy 5	Number of functions of the UAV	Number of functions of the UAV (scan only, delivery only, multirole)	No
Strategy 6	Percentage of UAVs with the same functionality in the swarm	Percentage of UAVs of the same functionality in the swarm	No
Strategy 7	Percentage of tasks the UAV can perform out of all tasks	Percentage of tasks the UAV can perform out of all survivors	No
Strategy 8	Remaining resources of the UAV (remaining task executions)	Remaining resources of the UAV (remaining task executions)	No
Strategy 9	Movement speed of the UAV	Movement speed of the UAV	No
Strategy 10	SEAD resource consumption rate of the UAV	Rescue resource consumption rate of the UAV	No
Strategy 11	Distance between tasks	Spatial distance between survivors	No
Strategy 12	Distance between task and all functionally compatible UAVs	Distance between survivor and all UAVs capable of its required task	No
Strategy 13	Percentage of UAVs in the swarm functionally aligned with the task	Percentage of UAVs in the swarm capable of the current survivor’s required task	No
Strategy 14	Time-window function value of the task	Time-window function value of the survivor’s task	No
Strategy 15	Resources required to complete the task	Resources required to complete the task	No
Strategy 16	Execution order of the task (recon, strike, assess)	Execution order of the task (scan/delivery)	No
Strategy 17	Heuristic value computed in the previous round	Heuristic value computed in the previous round	No
Strategy 18	Random constant between [0,1]	Random constant between [0,1]	No

This mapping process requires no modification to the algorithm’s source code or parameter settings; only the input feature definitions are updated to draw from survivor attributes and onboard sensor readings rather than target attributes and global truth tables. This demonstrates the generality of the CE&SG architecture across disparate robotic mission domains.

2. Integration of kinematic and collision constraints

The original PIHH formulation, as evaluated in the SEAD discrete-event simulations, treats UAVs as point masses capable of instantaneous movement between task locations. In the AirSim USAD environment, however, UAVs are subject to realistic kinematic constraints, including bounded acceleration, maximum velocity, and collision volumes. To adapt PIHH to these physical constraints without compromising its self-organizing strategy generation mechanism, we introduce a lightweight collision-aware heuristic modifier within the event-triggered meta-algorithm.

During the UAV selection phase of Algorithm 2, the heuristic value $H\left(U_{i}\right)$ computed by the self-attention mechanism is augmented with a collision-risk penalty term. The modified selection score is defined as:

$$H^{\prime }\left(U_{i}\right)=H\left(U_{i}\right)-\lambda \cdot C\left(U_{i}\right)$$ (26)

where $C\left(U_{i}\right)$ aggregates the reciprocal of the minimum predicted separation distance between $U_{i}$ and all other UAVs and static obstacles over a forward time horizon of τ=2.0 s. Specifically,

$$C\left(U_{i}\right)=\sum_{j\neq i}\frac{1}{d_{\mathrm{ij}}}+\sum_{o}\frac{1}{d_{\mathrm{io}}}$$ (27)

with $d_{\mathrm{ij}}$ denoting the estimated minimum distance between UAVs *i* and *j* given their current velocity vectors, and $d_{\mathrm{io}}$ denoting the distance to the nearest static obstacle. The scaling coefficient λ is set to 0.15 based on empirical tuning across a validation set of small-scale instances (*M* = 6, *N* = 3).

This modification effectively biases the strategy generation process away from task assignments that would precipitate congested or unsafe airspace configurations. Importantly, the penalty term is integrated solely at the heuristic evaluation stage; the underlying self-attention mechanism and fission–fusion interaction structure remain unchanged. This demonstrates that the CE&SG framework can natively incorporate physical safety considerations through lightweight, domain-specific adaptations without altering its core self-organizing logic.

The empirical results presented in the “Comparison with recent learning based methods” section confirm that this augmented PIHH deployment successfully navigates the USAD environment, achieving superior task efficiency and substantially reduced collision rates compared to both traditional hyper-heuristics and contemporary MARL methods. This validates the architectural flexibility of CE&SG: the strategy generation paradigm remains invariant across problem domains, while the perceptual interfaces and heuristic modifiers are adapted to the demands of high-fidelity robotic simulation.

3. Baseline implementation details

All comparative methods are evaluated under identical experimental conditions. The AirSim USAD environment provides each UAV with a 22-dimensional continuous observation vector comprising: UAV pose (6-d), proximity to nearest obstacles (6-d), target survivor information (8-d), and resource status (2-d). All methods output discrete UAV-task allocation decisions. Twenty independent random instances (*M* = 6/12/18, each with *N* = 0.5*M* UAVs) are used for training and evaluation, on a single NVIDIA RTX 3090 GPU. Table 11 summarizes the architecture, key hyperparameters, and USAD-specific adaptations for each method. All MARL baselines are trained using the configurations recommended in their original publications. All HH and meta-heuristic baselines use the parameter settings reported in their original publications. Where input/output dimensionalities differ between the original problem domain and USAD, only the input/output layers are remapped; core algorithmic logic and training hyperparameters remain unchanged.

**Table 11.** Baseline implementation details for USAD adaptation

Method	Architecture	Key hyperparameters	USAD-specific adaptation
DRL-HH	DDQN, 4-layer FC	Experience replay, *ε*-decay 1.0→0.01, *γ* = 0.99, 2,000 episodes	State: 22-d continuous observation; Action: *N* × *K* discrete allocation; Reward: completed tasks − 0.15×collisions
CBHH	Compass-guided MO-HH	*N* = 91, 500 iter, LLH = 10 gen, *τ* = 8, *K* = 6	8 discrete LLHs (nearest-first, urgency-first, best-match, etc.); Obj: max (task rate) + min (collisions)
RSOA	3-group meta-heuristic	*N* = 50, 500 iter, mature 40%/adol. 30%/infancy 30%, parameter-free	Rank-based discretization; individual = *N* × *M* priority vector; fitness = task count − collision penalty
E2E-MoE	MoE (N experts, 1-layer MLP each, ReLU+Softmax gate)	Adam lr = 0.01, *β* = (0.9,0.999), *λ* = 0.3, 5 × 10^5^ episodes	Input: 22-d state; experts = *N* (UAVs); *λ* adjusted 0.5→0.3 for collision weight
CTED-MAPPO	MAPPO + CTDE + LSTM critic	Actor: 300-64 Tanh; Critic: LSTM(20,20)-32-1 ReLU+LN; GAE(λ); 100-ep reset	Agent/UAV; obs: 22-d; Beta→velocity, Categorical→task; LSTM aggregates global state
DNC-A2C	A2C + DNC memory + PPO clip	Actor: 300-50-50; DNC: 256 × 64 mem, 3R/1W heads; lr = 1 × 10^−4^; batch = 20; 1,200 ep; *γ* = 0.95; *ε* = 0.1	DNC reads UAV task history (K-step S-A-R); GRU: collision risk prediction
HMRTA-RL	Attention encoder–decoder, MHA (*Z* = 8, *d* = 128) + GLU	REINFORCE + POMO baseline (*β* = 10); Adam lr = 1 × 10^−5^, decay 0.98/2,000 ep; Tmax = 100	Input: 22-d obs → graph features; Output: task index → UAV-task pair; CFM retained for training; test: Boltzmann (10 runs, best)
PIHH (ours)	Self-attention + dual-layer fission–fusion	*K* = 18 base strategies, *η* = 0.5, MaxIter = 50	Collision penalty augment [Eqs. (26) and (27)], *β* = 0.15; semantic remapping

### Theoretical property discussion

The discussions in this section are heuristic in nature and do not constitute formal mathematical proofs. They are intended to provide qualitative insight into the algorithm’s behavior under simplified modeling assumptions. Key assumptions include but are not limited to the following: (a) the state space is finite, guaranteed by discrete task and UAV counts; (b) the disturbance magnitude is bounded per Observation 2 below; and (c) the environmental reward distribution is stationary for the learning analysis. These assumptions may not hold in all real-world deployments, and the discussions below should accordingly be interpreted as qualitative property characterizations rather than rigorous guarantees. The analyses in the “Convergence behavior under static conditions”, “Stability characteristics under bounded disturbances”, “Learning dynamics of weight updates”, and “Computational complexity analysis” sections address convergence under static conditions, stable recovery under bounded disturbances, weight learning dynamics, and computational tractability. Several predictions align with experimental observations: rapid convergence (see the “Case study: Patterns of strategy evolution in SEAD” section), robust recovery under dynamic disturbances (Fig. 3), stable scalability (Fig. 4), and differentiated strategy activation across conditions (Fig. 5).

#### Convergence behavior under static conditions

This section examines the convergence behavior of the PIHH algorithm to a locally stable strategy set within a finite number of steps under static environments, arguing that it reaches a locally stable strategy set. We first define relevant notations and assumptions.

Definition 1 (state space)

Let *S* denote the set of all feasible task allocation schemes. Each scheme s∈S consists of UAV-task assignment pairs satisfying the constraints [Eqs. (8) to (15)]. According to Eq. (13), each UAV *U_i_* has a resource capacity *B_i_*; according to Eq. (12), the maximum travel distance is $d_{\mathrm{Max}}^{i}$. Thus, the number of tasks that a single UAV can execute is bounded by:

$$\begin{array}{c} \left|\pi_{i}\right|\leq \min\left(\frac{B_{i}}{\min_{m}r_{m}},\;\frac{d_{i}^{\mathrm{Max}}}{\min_{j}d\left(P_{i},P_{j}\right)}\right) \end{array}$$ (28)

where $\pi_{i}$ is the task sequence of UAV *U_i_*, *r_m_* is the resource requirement of one task, and $d\left(\cdot ,\;\cdot \right)$ denotes Euclidean distance. Since the number of UAVs *N* and the number of tasks *M* are both finite, we have $\left|S\right|<\infty$, i.e., the state space is finite.

Observation 1 (monotonicity of the objective function)

Let *S_k_* denote the allocation scheme after the *k*th main loop of Algorithm 2, and let *Z*(*S_k_*) denote the corresponding objective function value [Eq. (1)]. Then:

$$Z\left(S_{k+1}\right)\geq Z\left(S_{k}\right)$$ (29)

Justification: Each main loop of Algorithm 2 performs the following operations: a task *T*^#^ and a UAV *U*^#^ via Algorithm 1, and append *T*^#^ to the end of *U*^#^’s task queue (only if *U*^#^ has sufficient remaining resources to execute *T*^#^). Let $\Delta Z=Z\left(S_{k+1}\right)-Z\left(S_{k}\right)$. According to equation:

$$Z=W_{1}Z_{1}-W_{2}Z_{2}-W_{3}Z_{3}$$ (30)

where *Z*_1_ is the total reward of completed tasks, *Z*_2_ is the UAV usage cost, and *Z*_3_ is the time cost. The newly added task *T*^#^ contributes a reward $v_{T^{\#}}\cdot \lambda_{T^{\#}}\left(t\right)$ [Eqs. (3) and (4)], so $\Delta Z_{1}\geq 0$. The UAV usage cost *Z*_2_ is nondecreasing when a task is added, but since *Z*_2_ appears with a negative sign in the objective function, its contribution to ΔZ is nonpositive. However, the selection mechanism of Algorithm 2 (maximizing the heuristic value) ensures that a task is allocated only if its addition improves the overall objective value. Given that task rewards are nonnegative and a UAV is allocated a task only when its resources allow, we have ΔZ>0.

Key Property 1 (finite-step convergence behavior)

Under a static environment, Algorithm 2 terminates within a finite number of steps, and the terminal state *S*^#^ satisfies that there exists no feasible task $T_{e}\in G$ and UAV $U_{i}\in U$ such that assigning *T_e_* to *U_i_* improves the objective function value.

Justification: By Observation 1, the sequence {*Z*(*S_k_*)} is nondecreasing. Since the state space *S* is finite, the objective function *Z*(*S*) takes values from a finite set, so *Z*(*S_k_*) cannot increase indefinitely. Let MaxIter be the number of iterations when the algorithm terminates; then, *Z*(*S*_MaxIter_) is a local maximum. If there existed a feasible task *T_e_* and UAV *U_i_* such that assigning *T_e_* to *U_i_* would increase *Z*, the event-triggered mechanism of Algorithm 2 would detect this opportunity and perform the assignment, contradicting the fact that *K* is a termination step. Therefore, the terminal state satisfies the local optimality condition.

Convergence rate analysis

Let *M*_max_ be the maximum number of tasks that can be allocated. In the worst case, the algorithm may need to explore all states. However, the heuristic ranking mechanism in Algorithm 1 guides the search toward high-reward directions, resulting in an actual number of iterations much smaller than the state space size. Empirical observations (see the “Case study: Patterns of strategy evolution in SEAD” section) show that the algorithm typically stabilizes within 4 interaction rounds, validating the convergence efficiency.

#### Stability characteristics under bounded disturbances

This section analyzes the stability of the PIHH algorithm under dynamic disturbances. Drawing on the concept of input-to-state stability (ISS) from control theory, we argue that under finite disturbances, the system state tends to return to a stable strategy set adapted to the post-disturbance environment.

Definition 2 (system state)

Let $x_{k}\in S$ denote the system state (task allocation scheme) after the *k*th main loop. A dynamic disturbance δ∈Δ is defined as an event from the uncertainty set *D* [Eqs. (16) to (19)], including new target emergence (*d*_1_), task information error (*d*_2_), and UAV failure (*d*_3_).

Definition 3 (disturbance impact scope)

For a disturbance δ, define the affected task set $T_{\delta }$ and UAV set $U_{\delta }$. For example, when UAV *u* fails (*d*_3_), $U_{\delta }=\left\{u\right\}$, and all tasks previously assigned to *u* become unfinished.

Observation 2 (boundedness of local disturbance)

For any disturbance δ∈Δ, the number of affected UAVs satisfies $\left|U_{\delta }\right|\leq 1$, and the number of tasks already assigned to an affected UAV satisfies $\left|\pi_{u}\right|\leq M_{\max}<\infty$. Therefore, the magnitude of state change caused by a disturbance is bounded:

$$\left\|x_{k+1}-x_{k}\right\|\leq \max_{u\in U_{\delta }}\left|\pi_{u}\right|+T_{\delta }\leq C_{\delta }$$ (31)

where $C_{\delta }<\infty$ is a constant.

Key Property 2 (ISS characteristics)

Consider the system dynamics under disturbances:

$$x_{k+1}=F\left(x_{k},\;\delta_{k}\right)$$ (32)

where *F* denotes the composite mapping of Algorithm 2 and Algorithm 1, and $\delta_{k}\in \Delta$ is the disturbance that may occur at step *k*. If there exist a function β∈KL and a function γ∈K such that for all initial states *x*_0_ and disturbance sequences {$\delta_{k}$}:

$$\left\|x_{k}\right\|\leq \beta \left(\left\|x_{0}\right\|,\;k\right)+\gamma \left(\left\|\delta_{\left[0,\;k-1\right]}\right\|\right)$$ (33)

then the system is input-to-state stable.

Justification: According to the event-triggered mechanism of Algorithm 2, a disturbance only triggers local reallocation for affected UAVs and tasks. For the unaffected part, the system remains in a converged state (by Key Property 1). Therefore, the system dynamics can be decomposed as:

$$x_{k+1}=\Phi \left(x_{k}^{\left(u\right)},\;x_{k}^{\left(r\right)},\;\delta_{k}\right)$$ (34)

where $x_{k}^{\left(u\right)}$ is the state of the subsystem affected by the disturbance, and $x_{k}^{\left(r\right)}$ is the state of the remaining subsystem. By Key Property 1, there exists T<∞ such that $x_{k}^{\left(r\right)}$ converges to a local optimum for all k≥T. For $x_{k}^{\left(u\right)}$, by Observation 2, the scale of the disturbance impact is bounded, and the self-attention weight update rule in Algorithm 1 satisfies:

$$\left\|w_{Q}\left(k+1\right)-w_{Q}\left(k\right)\right\|\leq \eta \cdot \left\|w_{Q}\left(k\right)\right\|\cdot N\left(\text{winner}\right)$$ (35)

where η<1 is the learning rate, and $N\left(\cdot \right)$ is a Gaussian neighborhood function with range [0, 1]. Hence, the weight update is a contraction mapping, guaranteeing that $x_{k}^{\left(u\right)}$ converges to a new equilibrium within a finite number of steps. Combining the above, the system satisfies the ISS condition.

Remark 1 (upper bound on recovery time)

Let τ be the convergence time for a single interaction round. Then, the total recovery time after a disturbance satisfies:

$$T_{\text{recovery}}\leq \tau \cdot \left(\left|U_{\delta }\right|+\left|T_{\delta }\right|\right)\cdot \text{MaxIter}$$ (36)

where MaxIter is the maximum number of interactions in Algorithm 1. Since $\left|U_{\delta }\right|\leq 1$ and $\left|T_{\delta }\right|$ is typically a small constant, this upper bound is independent of the problem scale, consistent with the experimental observation in the “Case study: Patterns of strategy evolution in SEAD” section that recovery occurs within 4 interaction rounds.

#### Learning dynamics of weight updates

This section analyzes the learning mechanism of the PIHH algorithm, arguing that the update rule exhibits characteristics analogous to implicit Hebbian learning based on reward feedback, and discussing conditions under which the weight matrices exhibit convergent behavior as well as a quantitative characterization of learning efficiency.

1. Formalization of the learning rule

The learning in PIHH is embodied in the updates of 2 core matrices: the demand feature matrix *w_Q_* and the matching feature matrix *w_K_*. According to the update rule in Algorithm 3, when a strategy *x* is selected as the winner in an interaction round, the corresponding feature vectors are updated as follows:

$$\begin{array}{l} w_{Q}^{\mathrm{new}}=w_{Q}^{\mathrm{old}}+\alpha \cdot \theta \left(\text{winner}\right)\cdot \left(w_{Q}^{\text{target}}-w_{Q}^{\mathrm{old}}\right)\\ w_{K}^{\mathrm{new}}=w_{K}^{\mathrm{old}}+\alpha \cdot \theta \left(\text{winner}\right)\cdot \left(w_{K}^{\text{target}}-w_{K}^{\mathrm{old}}\right) \end{array}$$ (37)

where α is the learning rate (set to 0.5 in this paper), $\theta \left(\cdot \right)$ is a Gaussian neighborhood function (range [0,1]), and $w_{Q}^{\text{target}}$ and $w_{K}^{\text{target}}$ are the ideal features corresponding to the winner strategy (i.e., its own current features). This rule is equivalent to:

$$\begin{array}{l} \Delta w_{Q}=\alpha \cdot \theta \left(\text{winner}\right)\cdot \left(w_{Q}^{\text{target}}-w_{Q}^{\mathrm{old}}\right)\\ \Delta w_{K}=\alpha \cdot \theta \left(\text{winner}\right)\cdot \left(w_{K}^{\text{target}}-w_{K}^{\mathrm{old}}\right) \end{array}$$ (38)

Definition 4 (strategy reward)

Let the heuristic value generated by strategy *x* in an interaction be *H*(*x*), and the corresponding actual task allocation reward be $Z\left(x\right)=W_{1}\cdot \Delta Z_{1}-W_{2}\cdot \Delta Z_{2}-W_{3}\cdot \Delta Z_{3}$. Strategies with higher rewards have a higher probability of being selected in subsequent interactions.

Observation 3 (correlation between Hebbian update and reward)

There exists a positive correlation between the magnitude of the weight update and the reward of the winning strategy. Specifically, for any 2 strategies *x* and *y*, if *Z*(*x*) > *Z*(*y*), the winner is more likely to be *x*, and after the update, *w_Q_* and *w_K_* move toward the feature direction of *x*. Formally:

$$E\left[\Delta w_{Q}\left|Z\left(x\right)>Z\left(y\right)\right|\propto \alpha \cdot \theta \left(x\right)\cdot \left\|w_{Q}^{\text{target}}-w_{Q}^{\mathrm{old}}\right\|\right]$$ (39)

and this expectation increases with the reward difference *Z*(*x*) − *Z*(*y*). This reflects the reinforcement effect of successful strategies.

Justification: From the self-attention mechanism of Algorithm 1, the probability that strategy *x* is selected as the winner is positively correlated with its heuristic value *H*(*x*), which in turn is linked to the cumulative historical reward through weight updates. When *Z*(*x*) > *Z*(*y*), *x* tends to have a higher heuristic value in subsequent interactions, increasing its selection probability. The weight update direction is determined by the winner’s feature vector, so the conditional expectation moves toward *x*.

2. Convergent learning dynamics

Consider the weight updates as a discrete-time dynamical system:

$$\begin{array}{l} w_{Q}\left(k+1\right)=w_{Q}\left(k\right)+\alpha \cdot \theta \left(\text{winner}\left(k\right)\right)\cdot \left(w_{Q}^{\text{target}}\left(k\right)-w_{Q}\left(k\right)\right)\\ w_{K}\left(k+1\right)=w_{K}\left(k\right)+\alpha \cdot \theta \left(\text{winner}\left(k\right)\right)\cdot \left(w_{K}^{\text{target}}\left(k\right)-w_{K}\left(k\right)\right) \end{array}$$ (40)

Key Property 3 (observed convergence behavior of weights)

Assume that the distribution of strategy rewards in the environment is stationary. Then, the weight sequences {*w_Q_*(*k*), *w_K_*(*k*)} exhibit convergent behavior toward the strategy with the highest expected reward. This qualitative behavior is consistent with the experimental observations in the “Interpretable artifacts of strategy generation” and “Case study: Patterns of strategy evolution in SEAD” sections.

Justification: As a qualitative argument, consider the candidate Lyapunov function:

$$V\left(k\right)=\frac{1}{2}{\left\|w_{Q}\left(k\right)-w_{Q}^{\#}\right\|}^{2}+\frac{1}{2}{\left\|w_{K}\left(k\right)-w_{K}^{\#}\right\|}^{2}$$ (41)

where $w_{Q}^{\#}$ and $w_{K}^{\#}$ are the feature matrices of a stable strategy. Since the update rule contracts toward the winner’s feature direction, and because α<1 and $\theta \in \left[0,\;1\right]$, we have:

$$\begin{array}{c} \begin{array}{c} E\left[V\left(k+1\right)-V\left(k\right)|\;\text{history}\right]\leq \alpha \cdot \theta \left(\text{winner}\right)\cdot \left({\left\|w_{Q}\left(k\right)-w_{Q}^{\#}\right\|}^{2}+{\left\|w_{K}\left(k\right)-w_{K}^{\#}\right\|}^{2}\right)\leq 0 \end{array} \end{array}$$ (42)

Under the stationarity assumption on rewards, *V*(*k*) exhibits convergent behavior to a limit. If that limit corresponded to a suboptimal strategy, there would be a positive probability of moving toward a better strategy, contradicting convergence. Therefore, the limit point must be an attractor corresponding to the optimal strategy.

3. Learning efficiency analysis

Remark 2 (adaptive exploration–exploitation balance)

The convergence speed of the weights is determined by the learning rate α and the neighborhood function θ. In the initial phase, the winner strategy has not yet stabilized, and θ takes relatively large values (near the center of the Gaussian), resulting in large weight updates that enable rapid exploration. As iterations proceed and the winner stabilizes, θ decays with distance, the update magnitude diminishes, and the system enters a fine-tuning stage. This mechanism achieves an adaptive exploration–exploitation balance, consistent with the observation in the “Case study: Patterns of strategy evolution in SEAD” section that the weight of the random strategy (Strategy 18) peaks at the onset of dynamics and then rapidly decays.

Remark 3 (sample complexity)

Let the size of the strategy space be $\left|X\right|$ (in this paper, $\left|X\right|$ = 18). The expected number of environment interactions required to learn the optimal strategy is:

$$O\left(\frac{\left|X\right|}{\alpha \cdot \bar{\theta }}\log\frac{1}{\varepsilon }\right)$$ (43)

where $\bar{\theta }$ is the average neighborhood weight (which is relatively large in the initial stage), and ε is the convergence tolerance. Since the strategy set is small, α = 0.5, and $\bar{\theta }$ is close to 1 in the initial stage, PIHH can achieve learning with low sample complexity, aligning with the experimental observation that convergence occurs within a few interaction rounds.

We emphasize that Remark 3 provides an idealized upper bound under the stationarity assumption. In practice, when the reward distribution shifts due to environmental nonstationarity, the actual sample complexity may differ. The qualitative consistency with experimental observations should not be interpreted as a formal sample-complexity guarantee.

#### Computational complexity analysis

To comprehensively evaluate the computational efficiency of the PIHH algorithm, this section analyzes the complexity of its key components and compares it with the benchmark algorithms. Notation: *N* is the number of UAVs; *M* is the number of tasks. The total number *M*_total_ of jobs in a scenario is 3*M*; *K* is the number of base strategies. For the task-selection phase, *K*_task_ = 8 (Strategies 11 to 18). For the UAV-selection phase, *K*_UAV_ = 12 (Strategies 1 to 10, 17, and 18); *I* is the maximum number of interactions (MaxIter); *P* is the population size for training (NumPop).

The PIHH algorithm consists of 3 main components: the 2-layer self-attention network, the event-triggered meta-algorithm, and the offline training process. (a) A single execution of Algorithm 1 computes the self-attention weights for all pairs of strategies within the current layer, yielding a computational complexity of O(*K*^2^), where *K* denotes the number of strategies in the current layer (*K* = 18 for the full strategy set, *K*_task_ = 8 for task selection, *K*_UAV_ = 12 for UAV selection) rather than the number of robots *N*. The per-call cost of Algorithm 1 is therefore constant with respect to swarm size. (b) Algorithm 2: During online deployment, all *M*_total_ tasks must be allocated. For each allocation step, the algorithm traverses all UAVs to perform strategy interactions. The overall complexity is O(*M*_total_·*I*·*N*·$K_{\mathrm{UAV}}^{2}$). Since I and *K*_UAV_ are constants, this complexity is linear in the problem scale *N* and *M*_total_. (c) Algorithm 3: With a population size *P*, the training complexity is O(*P*·*M*_total_·*I*·*N*·$K_{\mathrm{UAV}}^{2}$). This one-time cost is incurred only before deployment and does not affect online execution.

To quantitatively demonstrate the computational efficiency advantage of PIHH, we compare its complexity with that of the 3 benchmark algorithms. The results are summarized in Table 12.

**Table 12.** Comparison of computational complexity. *G* is the number of generations, *D* is the number of local interactions, and *D*_tree_ is the average depth of the GP trees.

Algorithm	Complexity for a single instance	Remarks
CMA-ES	O(*G*·*P*·(*N*+*M*_total_)^3^)	Cubic complexity from covariance matrix decomposition (*D* = *M*_total_ under compact encoding); continuous encoding with rank-based decoding for discrete assignment; infeasible for real-time requirements in large-scale problems.
DCM	O(*M*_total_·*N*·*D*)	Linear complexity, but lack of global guidance leads to larger effective interactions *D*
GPHH	O(*G*·*P*·(*M*_total_ +*N*)·*D*_tree_)	Static tree structure incurs evolutionary overhead during online deployment
PIHH (Ours)	O(*M*_total_·*I*·*N*·*K*^2^)	Online complexity linear in *N* and *M*_total_; small constant factors

Based on the above analysis, the computational efficiency advantages of PIHH are reflected in 3 aspects: First, linear scalability. The online complexity of PIHH is linear in the problem scale, whereas the cubic complexity of CMA-ES makes it impractical for large-scale scenarios. Second, efficient coordination with global guidance. Compared to DCM, PIHH leverages a self-attention mechanism to incorporate global information, reducing ineffective interactions and task conflicts, leading to higher practical efficiency. Third, offline training with lightweight online deployment. Unlike GPHH, which requires population-based evolution during deployment, PIHH shifts the major computational burden to a one-time offline training phase. Online execution only involves simple matrix operations, avoiding the overhead of evolutionary operations.

Encoding and complexity derivation for CMA-ES baseline:

• Encoding and dimensional mapping. A brief clarification of the CMA-ES complexity formulation in Table 12 is warranted, as CMA-ES is inherently a continuous optimizer whose application to discrete task allocation requires an explicit encoding strategy. We adopt a real-valued vector encoding with rank-based decoding—a standard technique for applying continuous evolutionary algorithms to combinatorial optimization. Specifically, each candidate solution is a real vector x ∈ ℝ*^D^*, where *D* = *M*_total_ under a compact encoding: each element encodes the index of the UAV assigned to the corresponding task. In large-scale scenarios, the task count *M*_total_ typically dominates the UAV count N, hence *D* = *M*_total_ = O(max(*N*, *M*_total_)) = O(*N* + *M*_total_).
• Cubic complexity derivation. The cubic term (*N* + *M*_total_)^3^ originates from the eigendecomposition (or Cholesky factorization) of the *D* × *D* covariance matrix C, which is the computational bottleneck of each CMA-ES generation and costs O(*D*^3^) [56]. Substituting *D* = O(*N* + *M*_total_) yields O((*N* + *M*_total_)^3^) per generation. Multiplying by the number of generations *G* and population size *P* gives the total complexity O(*G* × *P* × (*N* + *M*_total_)^3^) listed in Table 12. For a representative large-scale instance (*N* = 250, *M*_total_ = 1,500, *D* ≈ 1,500), the covariance decomposition alone requires approximately 3.4 × 10^9^ floating-point operations per generation—rendering real-time replanning infeasible. In contrast, PIHH’s online complexity O(*M*_total_·*I*·*N*·*K*^2^) involves only small matrix operations (*K* = 18) and scales far more favorably.
• Methodological note on continuous-to-discrete encoding. Beyond the complexity analysis, we note that the continuous-to-discrete encoding introduces a further methodological limitation: multiple distinct real vectors may decode to the same discrete assignment, and Euclidean distances in ℝ*^D^* do not faithfully preserve meaningful differences between task assignments. This encoding gap can misdirect the covariance adaptation process, providing an algorithmic-level explanation—beyond raw computational cost—for the performance disparity between CMA-ES and PIHH observed in Table 12. Recent work on discrete-variable handling in CMA-ES variants, including margin-based correction methods [2], has made progress in mitigating this issue, though the fundamental tension between continuous optimization dynamics and discrete solution spaces remains.

In addition to performance comparison, we also recorded the average runtime of each algorithm during online deployment to assess real-time suitability. On the largest-scale instance (*N* = 250, *M* = 500), PIHH achieved an average runtime of 8.7 s, while CMA-ES, DCM, GPHH, and GPHH-EM required 142.3, 35.6, 18.4, and 19.1 s, respectively. Even after accounting for the computational gap between our experimental platform (Intel i7-12700) and typical onboard computers, the measured runtime of PIHH on the *N* = 50, *M* = 100 instance (approximately 1.2 s) scales to an estimated 6 to 12 s on resource-constrained hardware, which still satisfies the real-time replanning constraints of typical tactical missions. This confirms the feasibility of deploying PIHH on onboard robotic platforms.

From a memory footprint perspective, the storage requirements of PIHH’s self-attention mechanism are determined by the number of base strategies *K* and the strategy feature dimension *d*, independent of swarm size *N*. The per-robot memory footprint of the self-attention mechanism is approximately 10 kB: strategy feature vectors (*K* × *d* = 324 floats, ~1.3 kB), score matrix (~1.3 kB), weight matrix (~1.3 kB), and output buffers. All terms depend only on *K* and *d*, not on *N*. Intra-subgroup communication payload is 324 floats (~1.3 kB per exchange), and subgroup size is bounded by the affinity threshold. Compared with end-to-end MARL methods using high-dimensional embeddings (e.g., 128 dimensions × 8 heads), PIHH’s compact strategy representation (~1.3 kB) favors deployment on platforms such as Jetson Nano and Raspberry Pi 4.

In summary, PIHH achieves linear complexity with small constant factors, demonstrating good scalability and high online computational efficiency, making it suitable for large-scale, highly dynamic task allocation scenarios.

## Data Availability

Data are available within the article or the Supplementary Materials.
